# A comparison among three maximal mathematical models of the glucose-insulin system

**DOI:** 10.1371/journal.pone.0257789

**Published:** 2021-09-27

**Authors:** Marcello Pompa, Simona Panunzi, Alessandro Borri, Andrea De Gaetano

**Affiliations:** 1 CNR-IASI, Laboratorio di Biomatematica, Consiglio Nazionale delle Ricerche, Istituto di Analisi dei Sistemi ed Informatica, Rome, Italy; 2 Università Cattolica del Sacro Cuore Rome, Rome, Italy; 3 CNR-IRIB, Consiglio Nazionale delle Ricerche, Istituto per la Ricerca e l’Innovazione Biomedica Palermo, Palermo, Italy; Sapienza University of Rome: Universita degli Studi di Roma La Sapienza, ITALY

## Abstract

The most well-known and widely used mathematical representations of the physiology of a diabetic individual are the Sorensen and Hovorka models as well as the UVAPadova Simulator. While the Hovorka model and the UVAPadova Simulator only describe the glucose metabolism of a subject with type 1 diabetes, the Sorensen model was formulated to simulate the behaviour of both normal and diabetic individuals. The UVAPadova model is the most known model, accepted by the FDA, with a high level of complexity. The Hovorka model is the simplest of the three models, well documented and used primarily for the development of control algorithms. The Sorensen model is the most complete, even though some modifications were required both to the model equations (adding useful compartments for modelling subcutaneous insulin delivery) and to the parameter values. In the present work several simulated experiments, such as IVGTTs and OGTTs, were used as tools to compare the three formulations in order to establish to what extent increasing complexity translates into richer and more correct physiological behaviour. All the equations and parameters used for carrying out the simulations are provided.

## Introduction

Type 1 diabetes mellitus is a pathological condition in which blood glucose levels reach excessively high values because of absent or insufficient insulin production [1–3]. It occurs mainly in juveniles, due to a combination of genetic determinants and environmental factors [1–3]. The worldwide spread of the disease has reached the 8.5% of all diabetes cases in the world, with about 210,000 affected children in the US only [4, 5]. Type 2 diabetes mellitus (T2DM) individuals, instead, exhibits excessive blood glucose concentrations mainly due to the insufficient efficacy of circulating insulin to stimulate tissue glucose uptake (insulin resistance) and is correlated with obesity [6, 7]. T2DM affects more than 400 million people around the world and it is estimated to double within the next 10 years [8, 9], with the increasing in obesity, age and the population size of ethnic groups at higher risk [6]. While metformin, troglitazone, sulfonilureas and other oral hypoglycemic drugs are, together with an improved lifestyle with increased exercise and weight loss, the first treatment choice for most patients with Type 2 diabetes [8], individuals with Type 1 diabetes mellitus require exogenous insulin administrations to supply the lack of endogenous pancreatic insulin production. Insulin cannot be administered orally and must be delivered subcutaneously, either with discrete boli (multiple daily injections, MDI) or through insulin pumps. Recently, much research efforts has been addressed to the development of the so-called “artificial pancreas” (AP) [10–12], a system that includes an insulin pump connected to a continuous glucose monitor (CGM) and to a (possibly model-based) control algorithm: the pump delivers an insulin amount calculated on the basis of predicted glycemia. In this case, insulin is administered via a small cannula inserted through the skin into the subcutaneous tissue. Insulin is released as a basal continuous infusion at pre-programmed variable rates, which differ from individual to individual, and as insulin boli at mealtimes.

Different strategies, using different mathematical models needed to describe and predict the dynamics of glucose and insulin, exist for the development of the control algorithms embedded in the APs [13–16]. The accuracy of these models is important in successfully determining the optimal therapy, i.e. the appropriate insulin dose to deliver in order to avoid both hyperglycaemic and hypoglycaemic episodes. The algorithms to be embedded in the pumps must therefore be developed, *in-silico*, testing their performance over a wide variety of possible physiological situations against a good mathematical model of the glucose-insulin system, implemented in software. The availability of physiologically plausible mathematical models to be used as part of the control algorithm and as a tool for simulating and testing the system *in silico* is therefore critical for the development of safe and effective insulin infusion control algorithms. It is mandatory that these models are able to reproduce the correct physiological behaviour under different conditions and/or experimental procedures. It is to be noted that many mathematical models of the glucose-insulin system exist. Some of these may be relatively simple approximations, to be used in order to interpret specific clinical testing procedures and identifying specific parameters of interest from relatively small data sets. Others may be relatively complex representations of what is known about the different aspects of glycemic control, including meals, insulin and other hormones, several distribution compartments and so on. We will henceforth denote the simpler models as “compact” and the more extended, hypothetically complete models as “maximal”. What is needed for control algorithm development is therefore a physiologically correct maximal model.

In the present work we analyse and compare three such maximal models of the glucose/insulin system, all three of which have been used in the development of infusion control algorithms: the Hovorka model [17–19], the Sorensen model [20, 21] and the more recent UVAPadova model [22–27]. The UVAPadova implementation used in the present work refers to the S2017 model version [24], which is the latest updated version of the model.

The implemented version of the Sorensen model derives from the equations presented in his original PhD thesis [20], with the corrections introduced in [28] and modified to account for both (possible) subcutaneous administration of insulin and for the reduced insulin-sensitivity, which would be expected in a diabetic patient with respect to a normal individual [1]. The three models present different levels of complexity and a comparison among them gives the opportunity to understand to which extent increasing complexity translates into richer and more correct physiological behaviour.

## Methods

### Comparison among models simulations

In the present work three maximal models of the glucose/insulin system are compared based on glucose and insulin predictions following several simulated experiments. The models analysed are the Hovorka model [17–19], the Sorensen model [20, 21] and the UVAPadova model [22–27]. The Sorensen model is the most complex; it has been extensively described in the Author’s PhD thesis and has been thoroughly analyzed in a previous work [28], correcting some errors which were present in the original description by the Author. An extended version of the model, including a representation of the gastrointestinal tract, was also presented in [28], and is used in the present work to simulate experiments where glucose is orally administered. Sorensen’s model consists of three sub-models (one for glucose, one for insulin and one for glucagon) that describe the time-course of the variable concentrations in the brain, liver, heart and lungs, periphery (tissue and muscles), gut and kidney. In its original version, it includes pancreatic release of insulin, which in the present work is not considered, given our primary attention to applications for Type 1 Diabetes Mellitus (T1DM). For completeness, all equations are reported in the Appendix. The set of parameter values adopted by Sorensen in his original work is compatible with the normal physiological response to any type of simulated perturbation experiment. The peculiar conditions of T1DM are approximated by Sorensen introducing modifications to the original model. This modified version is the one adopted in the next section. On the contrary, the Hovorka model and the UVAPadova model were originally formulated to represent the physiological behaviour of T1DM individuals, and no modification is necessary for the purpose of the present work. The comparison among the three models was conducted in three steps:

- a series of in-silico experiments were set-up and the three models were compared in terms of insulin and glucose concentrations over time;
- the three models were adapted to observations of glucose and insulin from an Oral Glucose Tolerance Test performed on a normal individual: the procedure allowed the estimation of the amount of insulin that would be needed to be administered as a bolus in order to obtain the observed time courses, together with some other crucial model parameters. The estimation procedure followed a weighted least squared approach, with weights *ω*_*i*_(*i* = 1, …, *n*) the inverse of the squared expectations. The variance-covariance matrix of the estimates was obtained with a linear approximation of the model at the optimum by computing ${\hat{\sigma }}^{2}{\left({\hat{J}}^{T}{\hat{S}}^{-1}\hat{J}\right)}^{-1}$, where *J* is the Jacobian; *σ*^2^ × *S* is the variance-covariance matrix of the observed vector; *S* is a diagonal matrix with elements (*i*, *i*) equal to the squared expectations; ${\hat{\sigma }}^{2}$ is calculated as $\frac{1}{n-p}\sum_{i}\hat{\omega_{i}}\times {\left(y_{i}^{obs}-{\hat{y}}_{i}\right)}^{2}$, with *n* the number of observations and *p* the number of free parameters. The symbol ^ is used to indicate quantities computed at the optimum.
- the estimates obtained in the previous step were used to simulate subsequent two other OGTTs, with the administration of the same amount of glucose and insulin given in the first OGTT, with the aim of simulating glucose and insulin concentrations during one day as to mimic three meals.

#### The *in-silico* experiments

The implemented *in-silico* experiments are described as follows:

- *Intra Venous Glucose Tolerance Test (IVGTT)*: a continuous administration of basal insulin of 6.67 mU/min, in conjunction with 0.5 g/kg of glucose administered over 3 minutes.
- *IVGTT + insulin bolus*: a continuous administration of basal insulin of 6.67 mU/min, in conjunction with 0.5 g/kg of glucose administered over 3 minutes, accompanied by a bolus of 1000mU delivered in 1 minute.
- *OGTT + basal insulin administration*: an oral administration of 100 g of glucose in 1 minute was simulated together with a continuous administration of basal insulin of 6.67 mU/min.
- *OGTT + basal insulin + insulin bolus*: an OGTT of 100 g of glucose was administered over 1 minute with a continuous basal insulin delivery of 6.67 mU/min in combination with 1000 mU IVITT bolus in 1 minute.

All the above experiments were performed by setting the initial conditions (which also represent the steady state conditions) at a glucose concentration of 5 mmol/L with a basal insulin delivery of 6.67 mU/min for each model considered.

### The Sorensen T1DM model

The original version of the Sorensen model was adapted by the Author himself to represent a subject with T1DM. In this process of adapting normal physiology to impaired physiology, the model was modified by removing the pancreatic insulin secretion sub-model and fixing the scale of absolute concentrations of the metabolic source and sink functions in such a way that the diabetic response to any combination of circulating glucose, insulin and glucagon concentrations would have been the same as that of normal individuals subjected to similar conditions. The model adapted in this way therefore represents what would be a “normal” response in a subject with T1DM: for this reason it suffers from an important limitation in that it does not take into account the physiological abnormalities typically present in association with diabetes. In fact, the Author stated that the objective of modelling diabetes condition in the context of his work was “…to provide a basis for designing and assessing improved insulin therapies, and in particular for developing an insulin infusion algorithm for closed-loop insulin delivery based on blood glucose measurement.”. In this perspective, the comparison of the efficacy of different therapeutic regimens might be considered as largely independent of the details of the physiological model adopted: this is of course no longer true when comparing different models with one another.

A set of normal glucose and insulin concentrations at baseline (Table 1) must be adopted to calculate metabolic rates during diabetic model simulations. This stems from the fact that the post-absorption steady state in the insulin-treated diabetic subjects cannot be determined by the model parameters themselves (as is the case for simulations of normal subjects) as it is dependent on an external forcing function (*γ*_*IVI*_), which represents the input rate of peripheral venous insulin administration (the therapeutic regimen).

**Table 1:** Reference normal basal state glucose and insulin concentrations used to fix the concentration scales defining normal metabolic source and sink rates.

Reference normal basal concentrations	Used for calculation of
$G_{L}^{B}=101\frac{mg}{dl}$	Hepatic Glucose Uptake, *r*_*HGU*_ Hepatic Glucose Production, *r*_*HGP*_
$G_{PI}^{B}=86.8\frac{mg}{dl}$	Peripheral Glucose Uptake *r*_*PGU*_
$G_{H}^{B}=91.9\frac{mg}{dl}$	Pancreatic Glucagon Release *r*_*P*Γ*R*_
$I_{L}^{B}=21.4\frac{mU}{l}$	Hepatic Glucose Uptake *r*_*HGU*_ Hepatic Glucose Production *r*_*HGP*_
$I_{PI}^{B}=5.30\frac{mU}{l}$	Peripheral Glucose Uptake *r*_*PGU*_
$I_{H}^{B}=15.2\frac{mU}{l}$	Pancreatic Glucagon Release *r*_*P*Γ*R*_

In the case of T1DM modelling, an iterative method for variable initialization must be adopted. Assuming that the response of diabetic subjects to circulating glucose concentrations is the same as in the normal situation, and setting the steady-state values of local glucose and insulin concentrations to values compatible with normal physiology, the glucose concentrations that are at equilibrium with the basal insulin imposed by the external variable *γ*_*IVI*_ must be calculated.

The procedure is performed by Sorensen and is reported in the Appendix in the subsection “T1DM Sorensen Model initialization”.

The equations derived from the steady state conditions are the same as those obtained under normal conditions (model for normal subjects) except for the equation related to *I*_*H*0_, where the *r*_*PIR*_ function is set to 0 and the *γ*_*IVI*_ is included in the numerator:

$$\begin{array}{c} I_{H0}=\frac{\gamma_{IVI0}}{Q_{H}^{I}-Q_{L}^{I}\left(1-F_{LIC}\right)-Q_{K}^{I}\left(1-F_{KIC}\right)-Q_{P}^{I}\left(1-F_{PIC}\right)-Q_{B}^{I}} \end{array}$$ (1)

$$\begin{array}{c} I_{PV0}=I_{H0}\left(1-F_{PIC}\right) \end{array}$$ (2)

$$\begin{array}{c} I_{K0}=I_{H0}\left(1-F_{KIC}\right) \end{array}$$ (3)

$$\begin{array}{c} I_{L0}=I_{H0}\left(1-F_{LIC}\right) \end{array}$$ (4)

$$\begin{array}{c} I_{B0}=I_{H0} \end{array}$$ (5)

$$\begin{array}{c} I_{J0}=I_{H0} \end{array}$$ (6)

$$\begin{array}{c} I_{PI0}=I_{PV0}-\frac{Q_{P}^{I}T_{P}^{I}}{V_{PI}}\left(I_{H0}-I_{PV0}\right) \end{array}$$ (7)

All the equations of the Sorensen model are reported in the Appendix (subsection “The Sorensen Model”). As mentioned above, the *γ*_*IVI*_ input was introduced by Sorensen into the equation for *I*_*H*_ (insulin heart and lung compartment). This obviously represents a simplification, because in the treatment of patients with T1DM insulin is administered subcutaneously, via bolus or continuous infusion. To make the three models comparable, two subcutaneous compartments were therefore added to the revised Sorensen model (which already includes the gastro-intestinal compartment [28]). The model of the subcutaneous compartments used is equivalent to that present in the Hovorka model. The formulation adopted in the UVAPadova model is marginally more complicated: if the parameter *k*_*a*1_ is set to zero and the parameter *k*_*a*2_ is set to same value as the parameter *k*_*d*_, then the two formulations are equivalent. All the parameter values and descriptions are reported in Table 2.

**Table 2:** Sorensen model parameters.

Parameter	Units	Meaning	Value
$Q_{B}^{G}$	L/min	Vascular blood water flow rate for Brain (glucose-related)	0.59
$V_{BV}^{G}$	L	Distribution Volume of Glucose in Brain Vascular space	0.35
*V*_*BI*_	L	Distribution Volume of Brain Interstitial space	0.45
*T*_*B*_	min	Trans-capillary diffusion time constant for Brain	2.1
*r*_*BGU*_	mmol/min	Brain Glucose Uptake rate	0.388889
$Q_{L}^{G}$	L/min	Vascular blood water flow rate for Liver (glucose-related)	1.26
$Q_{K}^{G}$	L/min	Vascular blood water flow rate for Kidney (glucose-related)	1.01
$Q_{P}^{G}$	L/min	Vascular blood water flow rate for Peripheral tissues (glucose-related)	1.51
$Q_{H}^{G}$	L/min	Vascular blood water flow rate for Heart/lung (glucose-related)	4.37
*r*_*RBCU*_	mmol/min	Red Blood cell Glucose Uptake rate	0.0555556
$V_{H}^{G}$	L	Distribution Volume of Glucose in Heart/lung Vascular space	1.38
$Q_{J}^{G}$	L/min	Vascular blood water flow rate for Gut/Jejunum (glucose-related)	1.01
$V_{J}^{G}$	L	Distribution Volume of Glucose in Gut/Jejunum Vascular space	1.12
*r*_*JGU*_	mmol/min	Gut/Jejunal Glucose Uptake or utilization rate	0.233394
$Q_{A}^{G}$	L/min	Vascular blood water flow rate in hepatic Artery (glucose-related)	0.25
$V_{L}^{G}$	L	Distribution Volume of Glucose in Liver space	2.51
$V_{K}^{G}$	L	Distribution Volume of Glucose in Kidney space	0.66
$V_{PV}^{G}$	L	Distribution Volume of Glucose in Peripheral Vascular space	1.04
*V*_*PI*_	L	Distribution Volume of Peripheral Interstitial space	6.74
$T_{P}^{G}$	min	Trans-capillary diffusion time constant for Peripheral tissues (glucose-related)	5
$r_{PGU}^{B}$	mmol/min	Baseline rate of Peripheral Glucose Uptake	0.194444
$\beta_{PGU}^{0}$	#	PGU Insulin effect midpoint	0.703
$\beta_{PGU}^{1}$	#	PGU Insulin effect half-amplitude	0.652
$\beta_{PGU}^{2}$	#	PGU Insulin effect steepness	0.338
$\beta_{PGU}^{3}$	#	PGU Insulin effect shift	5.82
$\beta_{HGP}^{0}$	#	HGP gluCagon effect scale	2.7
$\beta_{HGP}^{1}$	#	HGP gluCagon scale	0.388852
*τ*_*C*_	min	Inverse of the decay rate for the glucagon-driven intensification of *f*_2_ Hepatic Glucose Uptake suppression	65
$\beta_{HGP}^{2}$	#	HGP Insulin effect midpoint	1.21
$\beta_{HGP}^{3}$	#	HGP Insulin effect half-amplitude	1.14
$\beta_{HGP}^{4}$	#	HGP Insulin effect steepness	1.66
$\beta_{HGP}^{5}$	#	HGP Insulin effect shift	0.887748
*τ*_*I*_	min	Inverse of the decay rate for the insulin-driven intensification of $M_{HGP}^{I}$ and $M_{HGU}^{I}$ (same for both)	25
$\beta_{HGP}^{6}$	#	HGP Glucose effect midpoint	1.0923
$\beta_{HGP}^{7}$	#	HGP Glucose effect half-amplitude	1.0846
$\beta_{HGP}^{8}$	#	HGP Glucose effect steepness	0.206667
$\beta_{HGP}^{9}$	#	HGP Glucose effect shift	0.504543
*r*_*HGP*0_	mmol/min	Baseline value of *r*_*HGP*_ at initial time (*t*_0_)	0.318611
$\beta_{HGU}^{0}$	#	HGU Insulin effect half-amplitude	2
$\beta_{HGU}^{1}$	#	HGU Insulin effect steepness	0.549306
$\beta_{HGU}^{2}$	#	HGP Glucose effect midpoint	5.66
$\beta_{HGU}^{3}$	#	HGP Glucose effect half-amplitude	5.66
$\beta_{HGU}^{4}$	#	HGP Glucose effect steepness	2.44
$\beta_{HGU}^{5}$	#	HGP Glucose effect shift	1.4783
*r*_*HGU*0_	mmol/min	Baseline value of *r*_*HGU*_ at initial time (*t*_0_)	0.111111
$\beta_{KGE}^{0}$	mmol/min	KGE Glucose effect midpoint	0.394444
$\beta_{KGE}^{1}$	mmol/min	KGE Glucose effect half-amplitude	0.394444
$\beta_{KGE}^{2}$	/mM	KGE Glucose effect steepness	0.198
$\beta_{KGE}^{3}$	mM	KGE Glucose effect shift, point of transition between tanh and linear regime	25.5556
$\beta_{KGE}^{4}$	mmol/min	KGE Glucose linear effect intercept	1.834
$\beta_{KGE}^{5}$	mmol/min/mM	KGE Glucose linear effect slope	0.0872
$Q_{B}^{I}$	L/min	Vascular blood water flow rate for Brain (insulin-related)	0.45
$V_{B}^{I}$	L	Distribution Volume of Insulin in Brain vascular space	0.26
$V_{H}^{I}$	L	Distribution Volume of Insulin in Heart/Lung vascular space	0.99
$Q_{L}^{I}$	L/min	Vascular blood water flow rate for Liver (insulin-related)	0.9
$Q_{K}^{I}$	L/min	Vascular blood water flow rate for Kidney (insulin-related)	0.72
$Q_{P}^{I}$	L/min	Vascular blood water flow rate for Periphery (insulin-related)	1.05
$Q_{H}^{I}$	L/min	Vascular blood water flow rate for Heart and Lungs (insulin-related)	3.12
$V_{J}^{I}$	L	Distribution Volume of Insulin in Gut Vascular space	0.94
$Q_{J}^{I}$	L/min	Vascular blood water flow rate for Gut (insulin-related)	0.72
$V_{L}^{I}$	L	Distribution Volume of Insulin in Liver Vascular space	1.14
$Q_{A}^{I}$	L/min	Vascular blood water flow rate in hepatic Artery (insulin-related)	0.18
*F*_*LIC*_	#	Fraction of insulin Liver clearance	0.459
*F*_*KIC*_	#	Fraction of insulin Kidney clearance	0.3
$V_{K}^{I}$	L	Distribution Volume of Insulin in Kidney Vascular space	0.51
$V_{PV}^{I}$	L	Distribution Volume of Insulin in Peripheral Vascular space	2.442
$T_{P}^{I}$	min	Trans-capillary diffusion time constant for Peripheral tissues (insulin-related)	20
*F*_*PIC*_	#	Fraction of insulin Periphery clearance	0.24
Γ_0_	pM	Starting value for glucagon	11.43
*r*_*MCC*_	L/min	Rate constant of glucagon clearance	0.91
*V*_Γ_	L	Glucagon distribution volume	11.31
$\beta_{PCR}^{0}$	#	PCR Glucose effect midpoint	2.93
$\beta_{PCR}^{1}$	#	PCR Glucose effect half-amplitude	2.1
$\beta_{PCR}^{2}$	#	PCR Glucose effect steepness	4.18
$\beta_{PCR}^{3}$	#	PCR Glucose effect shift	0.621325
$\beta_{PCR}^{4}$	#	PCR Insulin effect midpoint	1.31
$\beta_{PCR}^{5}$	#	PCR Insulin effect half-amplitude	0.61
$\beta_{PCR}^{5}$	#	PCR Insulin effect steepness	1.06
$\beta_{PCR}^{5}$	#	PCR Insulin effect shift	0.471419
*γ*_*IVG*0_	mmol/min	Intravenous Glucose Infusion starting value	0
*γ*_*SCI*0_	pmol/min	Subcutaneous Insulin Infusion starting value	0
*γ*_*IVI*0_	pmol/min	Intravenous Insulin Infusion starting value	46.746
*S*_0_	mmol	Baseline value of the stomach compartment at initial time (*t*_0_)	0
*k*_*js*_	1/min	Glucose transfer rate from Stomach to Jejunum compartment	0.01
*J*_0_	mmol	Baseline value of the jejunum compartment at initial time (*t*_0_)	0
*k*_*gj*_	1/min	Glucose transfer rate from Jejunum to Gut compartment	0.03672
*k*_*rj*_	1/min	Glucose transfer rate from Jejunum to Delay compartment	0.0351517
*R*_0_	mmol	Baseline value of the delay compartment at initial time (*t*_0_)	0
*k*_*lr*_	1/min	Glucose transfer rate from Delay to Ileum compartment	0.0289023
*L*_0_	mmol	Baseline value of the ileum compartment at initial time (*t*_0_)	0
*k*_*gl*_	1/min	Glucose transfer rate from Ileum to Gut compartment	0.0267142
*f*	#	Fraction of absorbed glucose	1
*R*_*oga*0_	mmol/min	Baseline value of *R*_*oga*_ at initial time (*t*_0_)	0
*r*_*PIR*0_	pmol/min	Baseline value of *r*_*PIR*_ at initial time (*t*_0_)	0
$G_{H}^{B}$	#	Reference normal basal state of glucose in the Heart/Lung compartment	5.105
$G_{PI}^{B}$	#	Reference normal basal state of glucose in the Peripheral Interstitial fluid space	4.822
$G_{L}^{B}$	#	Reference normal basal state of glucose in the Liver compartment	5.61
$I_{L}^{B}$	#	Reference normal basal state of insulin in the Liver compartment	150.01
$I_{PI}^{B}$	#	Reference normal basal state of insulin in the Peripheral Interstitial fluid space	37.128
$I_{H}^{B}$	#	Reference normal basal state of insulin in the Heart/Lung compartment	106.05
*τ*_*S*_	/min	Time constant for insulin absorption	55
*S*_10_	mU	Starting value of the amount of short-acting insulin in the compartment 1 starting value	Determined
*S*_20_	mU	Starting value of the amount of short-acting insulin in the compartment 2 starting value	Determined
*I*_*H*0_	pM	Starting value of *I*_*H*_ (insulin in the Heart/Lung compartment) at initial time (*t*_0_)	Determined
*I*_*PV*0_	pM	Starting value of *I*_*PV*_ (insulin in the Peripheral Vascular plasma space) at initial time (*t*_0_)	Determined
*I*_*K*0_	pM	Starting value of *I*_*K*_ (insulin in the Kidney compartment) at initial time (*t*_0_)	Determined
*I*_*B*0_	pM	Starting value of *I*_*B*_ (insulin in the Brain compartment) at initial time (*t*_0_)	Determined
*I*_*G*0_	pM	Starting value of *I*_*G*_ (insulin in the Gut compartment) at initial time (*t*_0_)	Determined
*I*_*PI*0_	pM	Starting value of *I*_*PI*_ (insulin in the Peripheral Interstitial fluid space) at initial time (*t*_0_)	Determined
*I*_*L*0_	pM	Starting value of *I*_*L*_ (insulin in the Liver compartment) at initial time (*t*_0_)	Determined
$I_{L0}^{N}$	#	Starting value of *I*_*NL*_ (normalized insulin in the Liver compartment) at initial time (*t*_0_)	Determined
$I_{PI0}^{N}$	#	Starting value of *I*_*NPI*_ (normalized insulin in the Peripheral Interstitial fluid space) at initial time (*t*_0_)	Determined
$I_{H0}^{N}$	#	Starting value of *I*_*NH*_ (normalized insulin in the Heart/Lung compartment) at initial time (*t*_0_)	Determined
*r*_*PIC*0_	pmol/min	Starting value of *r*_*PIC*_ at initial time (*t*_0_)	Determined
*M*_*PGU*0_	#	Starting value of *M*_*PGU*_ at initial time (*t*_0_)	Determined
*G*_*H*0_	mM	Starting value of *G*_*H*_ (glucose in the Heart/Lung compartment) at initial time (*t*_0_)	Determined
*G*_*K*0_	mM	Starting value of *G*_*K*_ (glucose in the Kidney compartment) at initial time (*t*_0_)	Determined
*G*_*L*0_	mM	Starting value of *G*_*L*_ (glucose in the Liver compartment) at initial time (*t*_0_)	Determined
*G*_*PV*0_	mM	Starting value of *G*_*PV*_ (glucose in the Peripheral Vascular blood water space) at initial time (*t*_0_)	Determined
*G*_*BV*0_	mM	Starting value of *G*_*BV*_ (glucose in the Brain Vascular space) at initial time (*t*_0_)	Determined
*G*_*J*0_	mM	Starting value of *G*_*J*_ (glucose in the Gut compartment) at initial time (*t*_0_)	Determined
*G*_*BI*0_	mM	Starting value of *G*_*BI*_ (glucose in the Brain Interstitial fluid space) at initial time (*t*_0_)	Determined
*G*_*PI*0_	mM	Starting value of *G*_*PI*_ (glucose in the Peripheral Interstitial fluid space) at initial time (*t*_0_)	Determined
$G_{H0}^{N}$	#	Starting value of *G*_*NH*_ (normalized glucose in the Heart/Lung compartment) at initial time (*t*_0_)	Determined
$G_{PI0}^{N}$	#	Starting value of *G*_*NPI*_ (normalized glucose in the Peripheral Interstitial fluid space) at initial time (*t*_0_)	Determined
$G_{L0}^{N}$	#	Starting value of *G*_*NL*_ (normalized glucose in the Liver compartment) at initial time (*t*_0_)	Determined
$M_{HGP0}^{I}$	#	Starting value of $M_{HGP}^{I}$ at initial time (*t*_0_)	Determined
$M_{HGPinf0}^{I}$	#	Starting value of $M_{HGPinf}^{I}$ at initial time (*t*_0_)	Determined
$M_{HGP0}^{G}$	#	Starting value of $M_{HGP}^{G}$ at initial time (*t*_0_)	Determined
$M_{HGU0}^{I}$	#	Starting value of $M_{HGU}^{I}$ at initial time (*t*_0_)	Determined
$M_{HGUinf0}^{I}$	#	Starting value of $M_{HGUinf}^{I}$ at initial time (*t*_0_)	Determined
$M_{HGU0}^{G}$	#	Starting value of $M_{HGU}^{G}$ at initial time (*t*_0_)	Determined
*r*_*KGE*0_	mmol/min	Starting value of *r*_*KGE*_ at initial time (*t*_0_)	Determined
*r*_*LIC*0_	pmol/min	Starting value of *r*_*LIC*_ at initial time (*t*_0_)	Determined
*r*_*KIC*0_	pmol/min	Starting value of *r*_*KIC*_ at initial time (*t*_0_)	Determined
$M_{P\Gamma R0}^{G}$	#	Starting value of $M_{P\Gamma R}^{G}$ at initial time (*t*_0_)	Determined
$M_{P\Gamma R0}^{I}$	#	Starting value of $M_{P\Gamma R}^{I}$ at initial time (*t*_0_)	Determined
$\Gamma_{0}^{N}$	#	Starting value of Γ_*N*_ at initial time (*t*_0_)	Determined
$M_{HGP0}^{\Gamma 0}$	#	Starting value of $M_{HGP}^{\Gamma 0}$ at initial time (*t*_0_)	Determined
*f*_20_	#	Starting value of *f*_2_ at initial time (*t*_0_)	Determined
$M_{HGP0}^{\Gamma }$	#	Starting value of $M_{HGP}^{\Gamma }$ at initial time (*t*_0_)	Determined
*r*_*PΓC*0_	pmol/min	Starting value of *r*_*PΓC*_ at initial time (*t*_0_)	Determined
$r_{P\Gamma R}^{B}$	pM/min	Baseline value of *r*_*PΓR*_	Determined
*r*_*PGU*0_	mmol/min	Starting value rate of the Peripheral Glucose Uptake	Determined
*r*_*HGP*0_	mmol/min	Starting value of the rate of Hepatic Glucose Production	Determined
*r*_*HGU*0_	mmol/min	Starting value of the rate of Hepatic Glucose Uptake	Determined
*r*_*PΓR*0_	pM	Starting value of the *r*_*PΓR*_ at initial time (*t*_0_)	Determined

BGU: Brain Glucose Uptake

GGU: Gut Glucose Utilization

HGP: Hepatic Glucose Production

HGU: Hepatic Glucose Uptake

KGE: Kidney Glucose Excretion

PGU: Peripheral Glucose Uptake

RBCU: Red Blood Cell Glucose Uptake

KIC: Kidney Insulin Clearance

LIC: Liver Insulin Clearance

PIC: Peripheral Insulin Clearance

PΓC: Plasma Glucagon Clearance

MΓC: Metabolic Glucagon Clearance

PΓR: Pancreatic Glucagon Release

### The Hovorka model

The Hovorka model includes two equations for glucose kinetics (amount of glucose in plasma and tissue). Input into the plasma compartment is determined by endogenous glucose production, which depends on plasma insulin concentration, and by absorption through the gastro-intestinal compartment. The equations and the values of the model parameters are shown in the Appendix. The term related to Endogenous Glucose Production (EGP), which appears in the final part of Eq [165], represents a linear inverse relationship between glucose production and insulin. This formulation could lead to negative EGP predictions in the presence of high levels of insulin concentrations or in correspondence with particular values of some parameters: a representation that incorporates a saturated effect would be more realistic. The gastro-intestinal tract is represented by two compartments: the *absorption* glucose compartment (*D*1), which is fed by the glucose equivalents of ingested carbohydrates, and the *conversion* compartment (*D*2) through which uptake of glucose occurs through a linear transfer to the plasma compartment. Insulin is released into the body by means of two subcutaneous compartments, *S*_1_ and *S*_2_, and then into the bloodstream which represents the plasma insulin compartment. All the values of parameters are shown in Table 3.

**Table 3:** Hovorka model parameters [17–19].

Parameter	Units	Meaning	Value
*G*_0_	mmol/L	The measurable blood glucose concentration starting value	5
*k*_12_	/min	Rate constant for transfer of glucose from the peripheral tissue into the blood stream	0.066
*BoW*	kg	Body weight	70
$F_{010}^{c}$	mmol/min	Insulin-indipendent glucose flux	0.679
*k*_*a*1_	/min	Deactivation rate constant	0.006
*k*_*a*2_	/min	Deactivation rate constant	0.06
*k*_*a*3_	/min	Deactivation rate constant	0.03
*k*_*b*1_	*L*/(*min*^2^*mU*)	Activation rate constant	3.072e-05
*k*_*b*2_	*L*/(*min*^2^*mU*)	Activation rate constant	4.92e-05
*k*_*b*3_	L/(min*mU)	Activation rate constant	0.00156
*M*_0_	mmol/min	Unit of meal	5.55556
*D*_0_	mmol/min	Oral CHO intake expressed as glucose equivalent starting value	0
*MealRate*_1_	g/min	Rate of CHO in the meal 1	10.625
*A*_*G*_	#	Factor expressing the utilization of CHO to glucose	0.8
*τ*_*D*_	min	Time constant	40
*u*_0_	mU/min	Rate of subcutaneous insulin infusion into compartment 1 starting value	6.68
*u*_1_	mU/min	Rate of subcutaneous insulin infusion into compartment 1 at time TimeInf11	1000
*u*_*basal*_	mU/min	Continuous rate of subcutaneous insulin infusion into compartment 1	6.68
*τ*_*S*_	min	Time constant for insulin absorption	55
*k*_*e*_	/min	The fractional elimination rate of insulin from the blood	0.138
*V*_*G*_	L	The distribution volume of the blood glucose compartment	Determined
*V*_*I*_	L	The distribution volume of the blood insulin compartment	Determined
*EGP*_0_	mmol/min	Endogenous release of glucose from the liver at the zero insulin concentration	Determined
*Q*_10_	mmol	Amount of glucose starting value	Determined
*F*_*R*0_	mmol/min	Renal excretion of glucose	Determined
*D*_10_	mmol	Glucose equivalence of CHO in the absorption compartment starting value	Determined
*D*_20_	mmol	Glucose in the conversion compartment starting value	Determined
*S*_10_	mU	Amount of short-acting insulin in the compartment 1 starting value	Determined
*S*_20_	mU	Amount of short-acting insulin in the compartment 2 starting value	Determined
*U*_*I*0_	mU/min	Insulin absorption rate into the blood starting value	Determined
*I*_0_	mU/L	Insulin concentration starting value	Determined
*U*_*G*0_	mmol/min	The exogenous input of glucose into blood stream from food absorption (the glucose absorption rate) starting value	Determined
*x*_10_	/min	The effect of insulin on distribution/transport of glucose starting value	Determined
*x*_20_	/min	The effect of insulin on glucose disposal starting value	Determined
*x*_30_	#	The (remote) effect of insulin on endogenous glucose production that released from liver starting value	Determined

### The UVAPadova model

The UVAPadova model used in the present work is the S2017 formulation presented in [24], which is an updated version of the original 2013 model [23]. This new formulation includes two new routes of insulin administration: inhaled insulin and intradermal insulin. Some parameters (*k*_*p*3_, *V*_*mx*_, *k*_*p*1_), which in the 2013 version were assumed to be constant, in the later version are made to be time-varying functions. Since their formulations have not been reported in the original work, in the present study we assumed piecewise constant functions for *k*_*p*3_ and *V*_*mx*_ from the inspection of Fig 2 in [24], whereas *k*_*p*1_ was set to a constant value. In addition, a new variable (*k*_*ir*_) is also added, which represents a decreasing factor of insulin dependent glucose utilization (*U*_*id*_). These latest modifications were included into the model to account for variability of metabolism over 24 hours.

The UVAPadova S2017 version makes use of two compartments for glucose (amounts of glucose respectively in plasma and tissues) and two compartments for insulin (amounts of insulin in plasma and liver). The glucose enters the system from the liver (*EGP*) and the gastro-intestinal tract [22]. Glucose exits the system due to renal elimination and due to glucose utilization, which in turn is divided into two terms: the *U*_*id*_ function, insulin-dependent utilization (whose correct formulation is reported in the 2013 version [23]) and the constant *U*_*ii*_, uptake of glucose by the brain and erythrocytes.

Insulin appears in plasma via three routes of administration: subcutaneous, inhaled and intra-dermal. The subcutaneous insulin sub-model is described in [25], while the intra-dermal model appears in [29] and the inhaled model is presented in [26]. The action of insulin on glucose is delayed by the introduction of variables that act both on EGP (decreasing as insulin increases) and on insulin-dependent glucose utilization *U*_*id*_ (increasing as insulin increases). Once again it should be noted that at high values of insulin concentration, the UVAPadova model would predict negative EGP’s.

The glucagon sub-model is composed of only one differential equation including:

- endogenous glucagon production (input);
- subcutaneous glucagon administration (input);
- glucagon elimination (output).

Glucagon affects glucose by enhancing its production (EGP increases with increasing glucagon levels in plasma); this effect also occurs through a delayed action. All the values of parameters and their sources are shown in Table 4.

**Table 4:** UVAPadova model parameters.

Parameter	Ref.	Units	Meaning	Value
*k*_1_	1	/min	Rate parameters	0.042
*k*_2_	1	/min	Rate parameters	0.071
*V*_*G*_	1	dL/kg	Distribution volume of glucose	1.49
*G*_*b*_	F	mg/dL	Glucose plasma starting value	90
*U*_*ii*_	1	mg/kg/min	Glucose uptake by the brain and erythrocytes	1
*m*_1_	5	/min	Rate parameters	0.356
*m*_2_	5	/min	Rate parameters	0.644
*m*_3_	5	/min	Liver degradation rate	0.534
*m*_4_	5	/min	Rate parameters	0.258
*V*_*I*_	1	L/kg	Distribution volume of insulin	0.044
*k*_*max*0_	3	/min	Maximum levels of gastric emptying rate starting value	0.03
*k*_*maxB*_	3	/min	Maximum levels of gastric emptying rate Breakfast	0.04
*k*_*maxL*_	3	/min	Maximum levels of gastric emptying rate Lunch	0.028
*k*_*maxD*_	3	/min	Maximum levels of gastric emptying rate Dinner	0.03
*Dose*	F	mg	Initial glucose Dose	100000
*k*_*abs*0_	3	/min	Rate constant of intestinal absorption starting value	0.147
*k*_*absBL*_	3	/min	Rate constant of intestinal absorption Breakfast and Lunch	0.13
*k*_*absD*_	3	/min	Rate constant of intestinal absorption Dinner	0.147
*f*	F	#	Fraction of glucose absorbed	0.7
*BW*	F	kg	Body weight	70
*k*_*min*0_	3	/min	Minimum levels of gastric emptying rate starting value	0.008
*k*_*minB*_	3	/min	Minimum levels of gastric emptying rate Breakfast	0.015
*k*_*minL*_	3	/min	Minimum levels of gastric emptying rate Lunch	0.01
*k*_*minD*_	3	/min	Minimum levels of gastric emptying rate Dinner	0.008
*b*	1	#	Fraction of dose corresponding to the flexes of gastric emptying curve	0.68
*c*	1	#	Fraction of dose corresponding to the flexes of gastric emptying curve	0.09
*Q*_*sto*0_	3	mg	Glucose into the Stomach starting value	0
*Q*_*sto*10_	3	mg	Glucose into the Solid Stomach compartment starting value	0
*Q*_*sto*20_	3	mg	Glucose into the Liquid Stomach compartment starting value	0
*Q*_*gut*0_	3	mg	Glucose into the Gut compartment starting value	0
*Ra*_*meal*0_	3	mg/kg/min	Rate of appearance of the meal starting value	0
*EGP*0	F	mg/kg/min	Endogenous Glucose Production starting value	2.4
*k*_*p*2_	1	/min	Hepatic glucose effetiveness	0.0007
*k*_*p*30_	3	mg/kg/min/pmol/L	Hepatic insulin sensitivity starting value	0.014
*k*_*p*3*B*_	3	mg/kg/min/pmol/L	Hepatic insulin sensitivity Breakfast	0.015
*k*_*p*3*LD*_	3	mg/kg/min/pmol/L	Hepatic insulin sensitivity Lunch and Dinner	0.014
*ξ*	C	mg/kg/min/ng/L	Hepatic responsivity to glucagon	0.013
*k*_*i*_	1	/min	Rate parameter accounting for delay between insulin signal and insulin action	0.0066
*k*_*H*_	C	/min	Inverse of time delay between glucagon concentration and action	0.009
$X_{0}^{H}$	3	ng/L	Delayed glucagon action on EGP starting value	0
*X*_0_	3	pmol/L	Insulin action on the glucose utilization starting value	0
*G*_*th*_	C	mg/dL	Hypoglycemic threshold	60
*V*_*m*0_	1	mg/kg/min	Rate parameter	4.65
*V*_*mx*0_	3	mg/kg/min/pmol/L	Insulin sensitivity	0.058
*V*_*mxB*_	3	mg/kg/min/pmol/L	Insulin sensitivity Breakfast	0.051
*V*_*mxLD*_	3	mg/kg/min/pmol/L	Insulin sensitivity Lunch and Dinner	0.058
*risk*_0_	F	#	Blood glucose risk function starting value	0
*r*_1_	C	#	Risk parameter 1	1.5
*r*_2_	C	#	Risk parameter 2	0.8
*K*_*m*0_	1	mg/kg	Glucose mass appearing in Michaelis-Menten relation	466.21
*p*_2*U*_	1	/min	Rate constant of insulin action on the peripheral glucose utilization	0.084
*k*_*e*1_	1	/min	Glomerular filtration rate	0.0007
*k*_*e*2_	1	mg/kg	Renal threshold of the glucose	269
*k*_*a*1_	2	/min	Rate constant of non-monomeric insulin absorption	0.0018
*k*_*a*2_	2	/min	Rate constant of monomeric insulin absorption	0.0182
*k*_*d*_	2	/min	Rate constant of insulin dissociation	0.0164
*k*_*aIih*_	5	/min	Rate constant	0.026
*F*_*Iih*_	5	/min	Rate constant	0.14
*I*_*ihss*_	5	pmol/kg	Inhaled insulin starting value	0
*T*_*s*_	F	min	Time constant	1
*n*	C	/min	Clearance rate	0.01
*H*_*b*_	F	ng/L	Glucagon plasma concentration starting value	58
*ρ*	C	/min	Rate parameter accounting for delay between static glucagon secretion and plasma glucose	0.86
*σ*	C	ng/L/min/mg/dL/pmol	Responsivity of alpha cells for glucose level	0.01
*δ*	C	ng/L*mg/dL	Responsivity of alpha cells for glucose rate of change	0.98
$SR_{Hb}^{d}$	4	ng/L/min	Glucagon secretion component 2 starting value	0
*k*_*h*1_	F	/min	Rate parameter describing subcutaneous glucagon kinetics 1	0
*k*_*h*2_	F	/min	Rate parameter describing subcutaneous glucagon kinetics 2	0
*k*_*h*3_	F	/min	Rate parameter describing subcutaneous glucagon kinetics 3	0
*H*_*sc*1*b*_	4	ng/L	Glucagon subcutaneous concentration 1 starting value	0
*H*_*sc*2*b*_	4	ng/L	Glucagon subcutaneous concentration 2 starting value	0
*Ra*_*Hb*_	4	ng/L/min	Rate of appearance of the glucagon starting value	0
*u*_*sc*0_	F	pmol/kg/min	Exogenous insulin infusion rate	0.667
*u*_*scBolo*_	F	pmol/kg/min	Exogenous insulin infusion rate bolus at the breakfast time	100
*u*_*scBasal*_	F	pmol/kg/min	Exogenous insulin infusion rate at the basal condition	0.667
*u*_*ih*_	F	pmol/kg/min	Inhaled insulin infusion rate	0
*G*_*pb*_	D	mg/kg	Glucose plasma concentration starting value	Determined
*E*_0_	D	mg/kg/min	Renal excretion starting value	Determined
*I*_*pb*_	D	pmol/kg	Insulin plasma concentration starting value	Determined
*I*_*b*_	D	pM	Insulin plasma starting value	Determined
$X_{0}^{L}$	D	pM	Delayed insulin action in the liver starting value	Determined
*k*_*p*1_	D	mg/kg/min	Extrapolated EGP at zero glucose and insulin	Determined
*G*_*tb*_	D	mg/kg	Glucose tissue concentration starting value	Determined
*U*_*id*0_	D	mg/kg/min	Insulin-dependent utilization starting value	Determined
*k*_*ir*_	D	#	Decrease factor for insulin-dependent glucose utilization	Determined
*I*_*lb*_	D	pmol/kg	Insulin liver concentration starting value	Determined
*α*	D	/mg	Constant	Determined
*β*	D	/mg	Constant	Determined
*k*_*empt*0_	D	/min	Emptying rate of the Stomach starting value	Determined
*f*_2_	D	#	Function 2	Determined
$I_{0}^{\prime }$	D	pM	Delayed insulin starting value	Determined
*I*_*sc*1*ss*_	D	pmol/kg	Amount of non-monomeric insulin in the subcutaneous space starting value	Determined
*I*_*sc*2*ss*_	D	pmol/kg	Amount of monomeric insulin in the subcutaneous space starting value	Determined
*G*_*b*_	D	mg/dL	Subcutaneous glucose starting value	Determined
*Ra*_*Isc*0_	D	pmol/kg/min	Subcutaneous insulin kinetics starting value	Determined
*Ra*_*Iih*0_	D	pmol/kg/min	Inhaled insulin kinetics starting value	Determined
*Ra*_*I*_	D	pmol/kg/min	External insulin rate of appearance starting value	Determined
$SR_{H}^{b}$	D	ng/L/min	Glucagon secretion starting value	Determined
$SR_{Hb}^{S}$	D	ng/L/min	Glucagon secretion component 1 starting value	Determined

1: Meal Simulation Model of the Glucose-Insulin System (2007) [22]

2: GIM, Simulation Software of Meal Glucose–Insulin Model (2007) [25]

3: One-Day Bayesian Cloning of Type 1 Diabetes Subjects: Towards a Single-Day UVA/Padova Type 1 Diabetes Simulator (2016) [27]

4: The UVA/Padova Type I Diabetes Simulator Goes From Single Meal to Single Day [24]

5: Improving Efficacy of Inhaled Technosphere Insulin (Afrezza) by Postmeal Dosing: In-silico Clinical Trial with the University of Virginia/Padova Type 1 Diabetes Simulator [26]

F: Fixed

D: Determined

C: Calibrated

## Results

**Fig 1:**
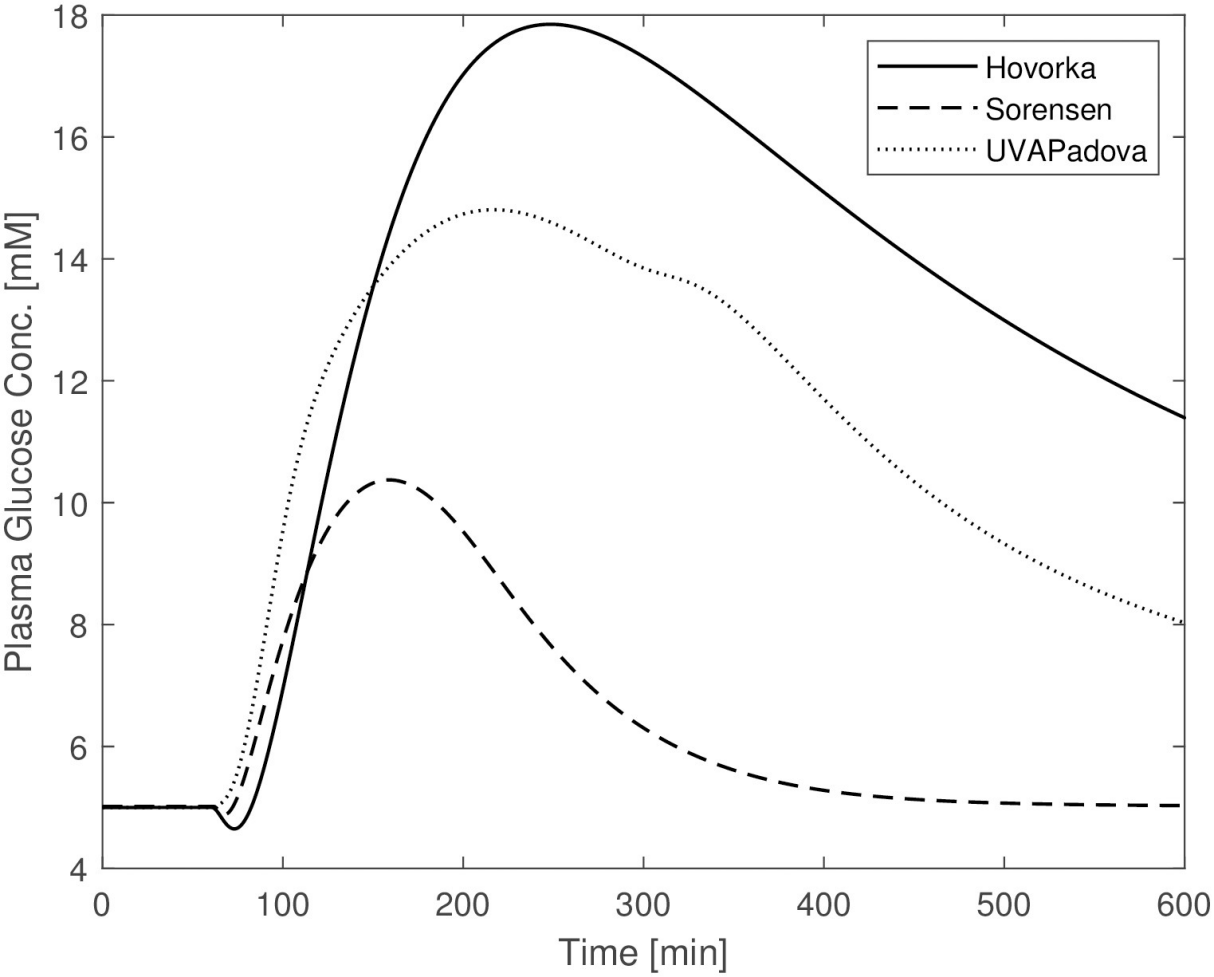
Blood glucose concentrations from Hovorka (solid line), Sorensen (dashed line) and UVAPadova (dotted line) models with the original Sorensen model, during an in-silico OGTT experiment in conjunction with an insulin bolus.

A first set of simulations showed that without making any change to the Sorensen model parameter values, the model is insufficient to predict reasonable time courses of blood glucose concentrations in diabetic subjects undergoing an OGTT: the curve reaches a maximum glycemia of 10 mM, a much lower value than that observed with the other two formulations (18 mM) and presumably observed in such patients; also, the time required for plasma glucose to return to its basal value is approximately 300 min, about half the time required for the Hovorka and UVAPadova models. One reason for the observed divergences from the expected behaviour relies on the assumption that, apart from the defect in insulin production, the physiology of a diabetic individual is otherwise the same as that of a normal individual. This assumption represents an understandable simplification in the absence of further information, but diabetic people are actually known to suffer from reduced insulin sensitivity as well [1], and avoiding to consider diabetic insulin resistance could lead to misleading results. In Fig 1 it can be seen that glucose concentrations forecasts by the Sorensen model differ substantially from those by the other two models. This suggests the need to modify the values of those Sorensen model parameters involved in the description of insulin-dependent glucose uptake. In order to check whether Sorensen’s model was qualitatively different from the other two models, parameter values were estimated for Sorensen’s model by adapting its predictions to the corresponding Hovorka and UVAPadova predicted time-courses for an OGTT plus insulin experiment (*OGTT + basal insulin + insulin bolus* in-silico experiment). In order to obtain comparable predictions, three steps were followed:

1. The peripheral venous insulin volume ($V_{PV}^{I}$) of the Sorensen model was increased to initialize steady-state insulinemia to a value as close as possible to the initial insulin concentrations observed for the Hovorka and UVAPadova models under the same basal insulin infusion conditions. Parameters *F*_*PIC*_ and *F*_*LIC*_, which represent fractional peripheral and hepatic insulin clearance, respectively, were also increased.
2. Plasma glucose concentrations, after oral administration of 100g of glucose without insulin delivery, were simulated with the Hovorka and Sorensen models and then compared to determine the values of the Sorensen parameters involved in the description of insulin-independent glucose production and elimination. The parameters and functions involved in the two processes are $r_{HGP}^{B}$ (the constant basal glucose production), *r*_*JGU*_ (the constant rate of intestinal glucose utilization) and $M_{HGP}^{G}$ (the hepatic glucose production) depending on parameters *β*_0*HGP*_, *β*_1*HGP*_ and *β*_2*HGP*_, which were all reduced to make the liver less sensitive to circulating glucose concentrations.
3. In the final step the plasma glucose concentrations from the Sorensen model, following an oral administration of 100g of glucose in combination with both an insulin infusion and an insulin bolus, were made as close as possible to the Hovorka time courses, by modifying the parameters involved in insulin-dependent glucose uptake ($M_{PGU}^{I}$). The modified parameters were *β*_0*PGU*_ and *β*_1*PGU*_, both decreased in order to reflect reduced peripheral tissue sensitivity to insulin, as expected in diabetic subjects.

**Fig 2:**
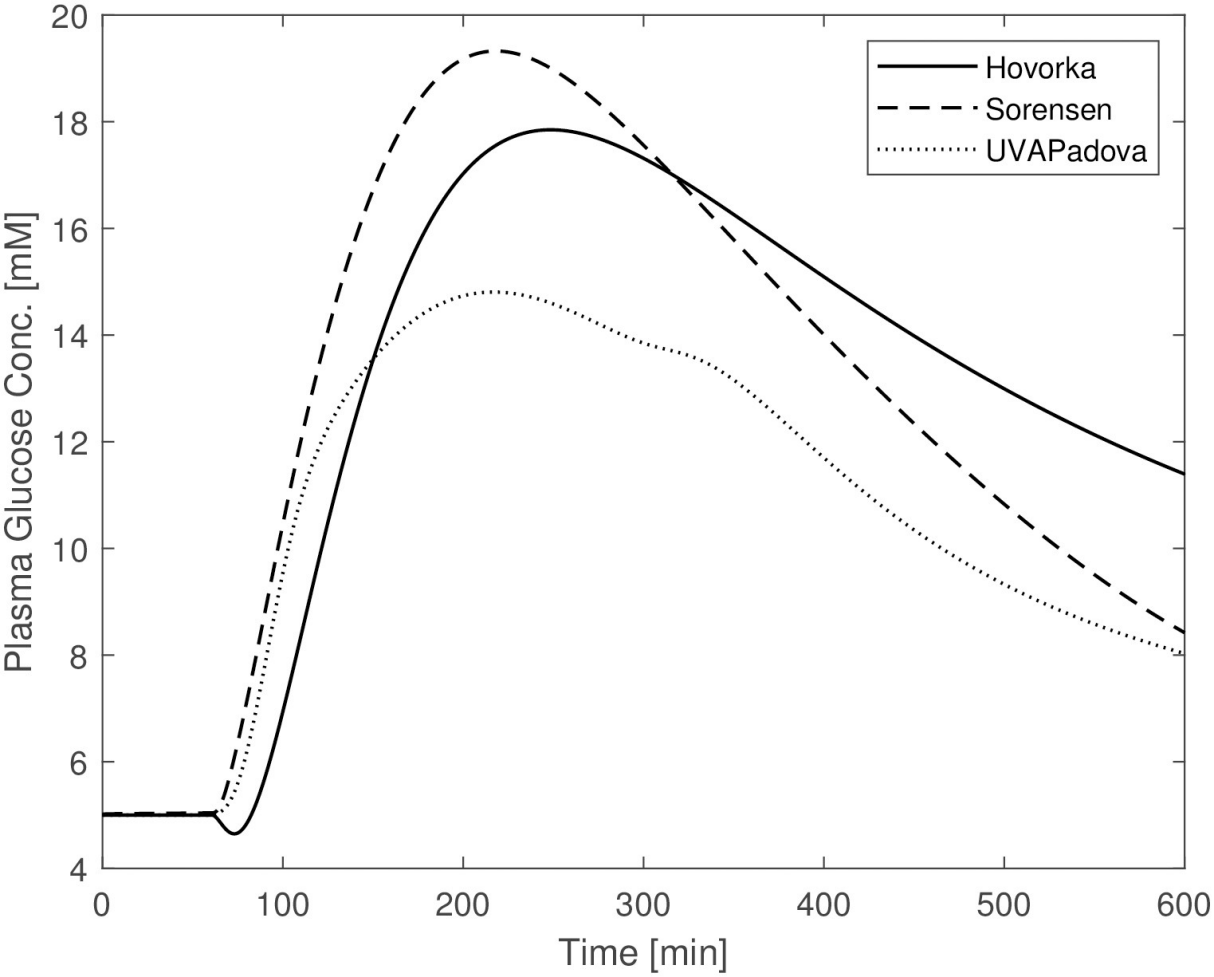
Blood glucose concentrations from the Hovorka (solid line), Sorensen (dashed line) and UVAPadova (dotted line) models with the modified Sorensen model, during an in-silico OGTT experiment in conjunction with an insulin bolus.

Fig 2 shows the plasma glucose concentrations resulting from the three models following the changes described above. The original and the modified parameter values are reported in Table 5 (“(before)” and “(after)” columns respectively). In this figure the predictions from the Sorensen model are comparable with those obtained under the Hovorka and UVAPadova model, exhibiting similar maximum concentrations despite a faster return to baseline conditions. Similar time courses were obtained by slightly modifying parameters *F*_*PIC*_ and *F*_*LIC*_ but by tripling $V_{PV}^{I}$ that was set to a value very close to that assumed by UVA/Padova. Large changes were also necessary for the parameters involved in the insulin-dependent glucose utilization which were decreased by about ten times. This seems however to be reasonable for a diabetic individual.

**Table 5:** Results of the set up calibration (trial and error) procedure to make the simulation of the Sorensen model comparable with the simulations of the Hovorka and UVAPadova formulations.

	Before	After
*F*_*PIC*_(#)	0.150	0.240
*F*_*LIC*_(#)	0.400	0.459
$V_{PV}^{I}\left(L\right)$	0.740	2.442
$r_{HGP}^{B}\left(\frac{mmol}{min}\right)$	0.861	0.319
$r_{JGU}\left(\frac{mmol}{min}\right)$	0.111	0.233
*β*_0*HGP*_(#)^1^	1.42	1.092
*β*_1*HGP*_(#)^1^	1.41	1.085
*β*_2*HGP*_(#)^1^	0.62	0.2067
*β*_0*PGU*_(#)^2^	7.03	0.703
*β*_1*PGU*_(#)^2^	6.52	0.652

^1^: $M_{HGP}^{G}=\beta_{0HGP}-\beta_{1HGP}tanh\left[\beta_{2HGP}\left(G_{L}^{N}-\beta_{3HGP}\right)\right]$

^2^: $M_{PGU}^{I}=\beta_{0PGU}+\beta_{1PGU}tanh\left[\beta_{2PGU}\left(I_{PI}^{N}-\beta_{3PGU}\right)\right]$

**Fig 3:**
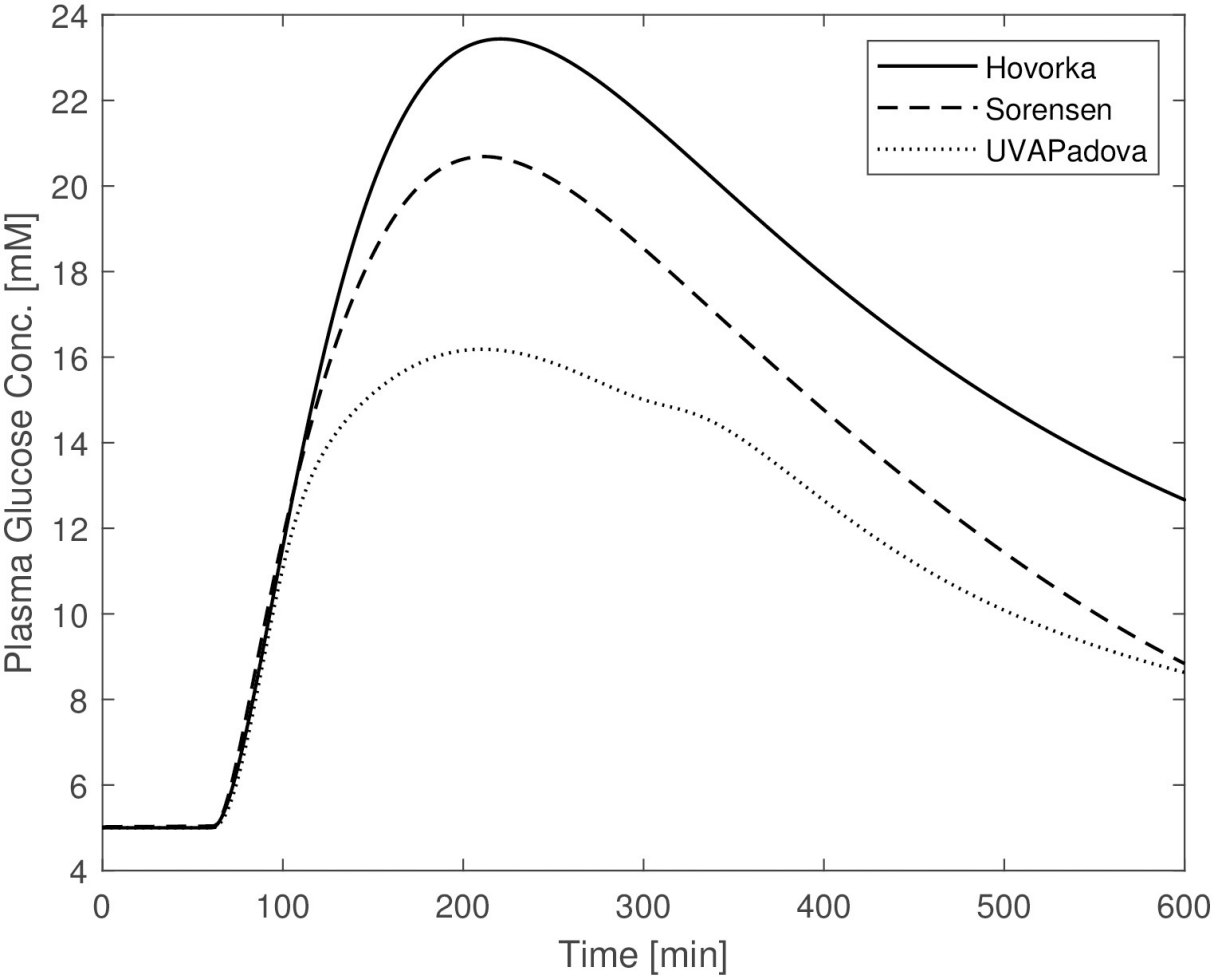
Blood glucose concentrations from the Hovorka (solid line), Sorensen (dashed line) and UVAPadova (dotted line) models, during the in-silico OGTT experiment.

**Fig 4:**
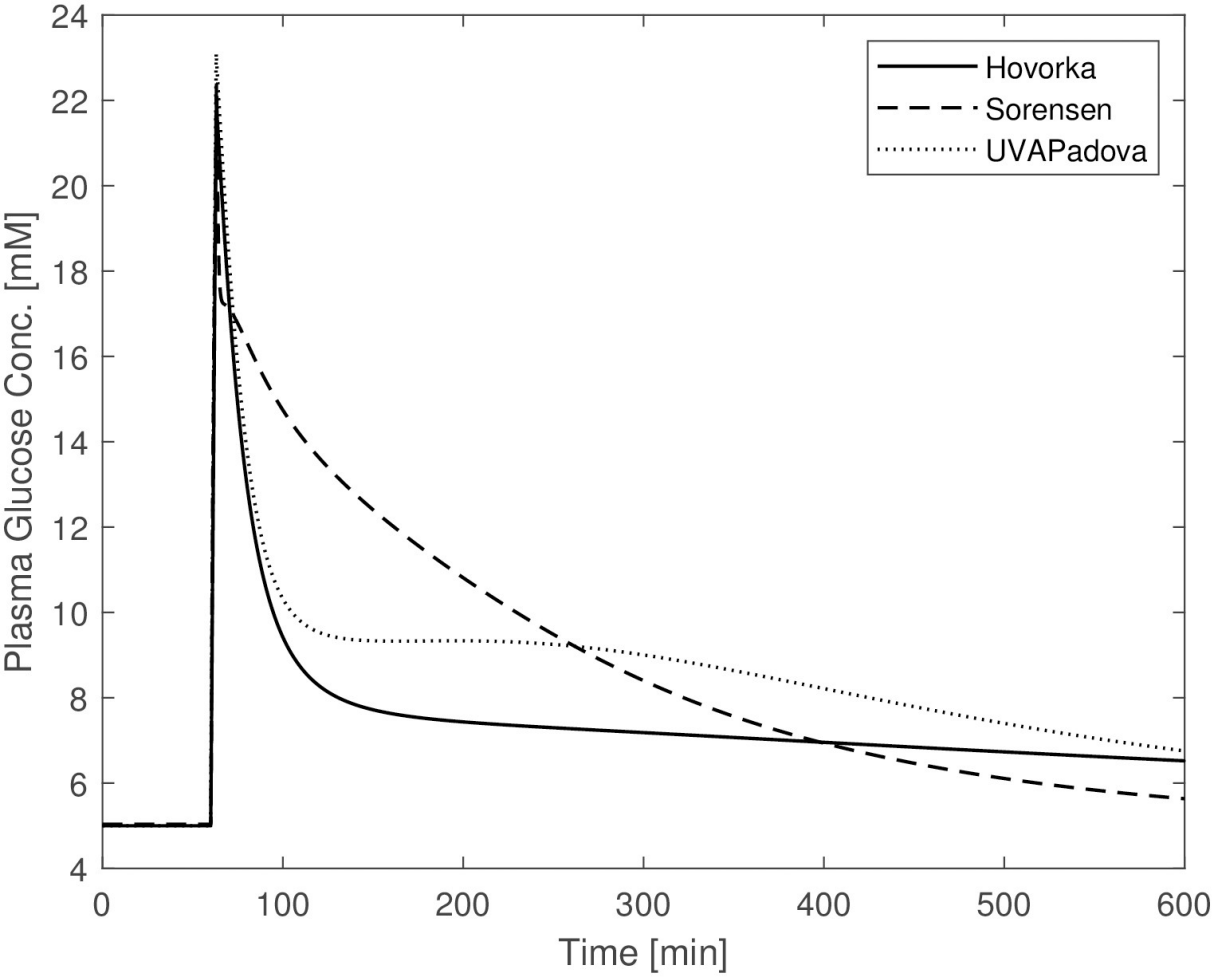
Blood glucose concentrations from the Hovorka (solid line), Sorensen (dashed line) and UVAPadova (dotted line) models, during the in-silico IVGTT experiment in conjunction with an insulin bolus.

**Fig 5:**
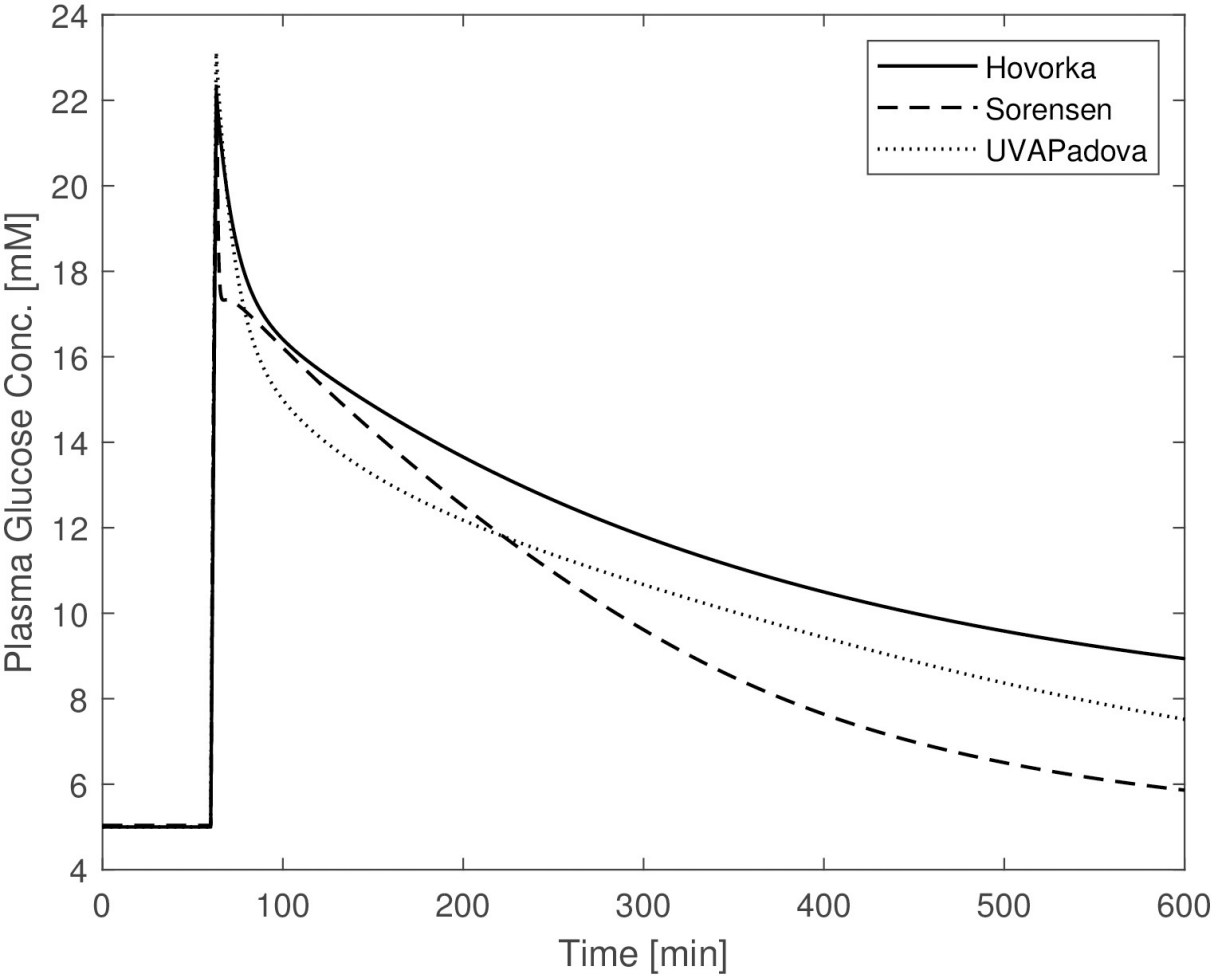
Blood glucose concentrations from the Hovorka (solid line), Sorensen (dashed line) and UVAPadova (dotted line) models, during the in-silico IVGTT experiment.

The other planned simulations, reported in the subsection “**The in-silico experiments**”, are shown in Figs 3–5. The figures show a similar behaviour of the three models with slight divergences: the Hovorka time courses differ from the trends observed for the Sorensen and UVAPadova models in the OGTT experiment without insulin administration (Fig 3); Sorensen’s predictions deviate from the other two in the IVGTT experiment (Fig 4). In the latter figure, the Sorensen model shows reduced insulin sensitivity compared to the other two formulations, exhibiting a much slower return to the basal values, more in line with the profile of a diabetic individual.

### OGTT model fitting

The three models were compared in terms of their ability to adapt to observed glucose concentrations from a normal individual undergoing an OGTT with the administration of 100 g glucose. Data were taken from Sorensen’s PhD thesis [20]. The fitting procedure was performed by minimizing the sum of the weighted squared residuals (weighted least-squares estimation, WLS, with weights the inverse of the squared expectations). The choice to use real data from a normal individual derives from the unavailability in the literature of OGTT data from diabetic subjects, since OGTT is not a standard procedure performed on diabetic people. It should be underscored that model parameters needed to be assessed numerically via fitting, because the parameter values used in the simulations above (representing the response of a diabetic individual), were inadequate to represent the physiological behaviour of normal subjects, who show different rates of absorption and production of glucose.

For each of the three models the fitting procedure allowed the estimation, among other things, of the amount of insulin administered as a bolus; the basal insulin was instead determined in such a way that all three models started (i.e. at time zero, before the glucose and insulin bolus administrations) from the same level of glucose concentration.

**Fig 6:**
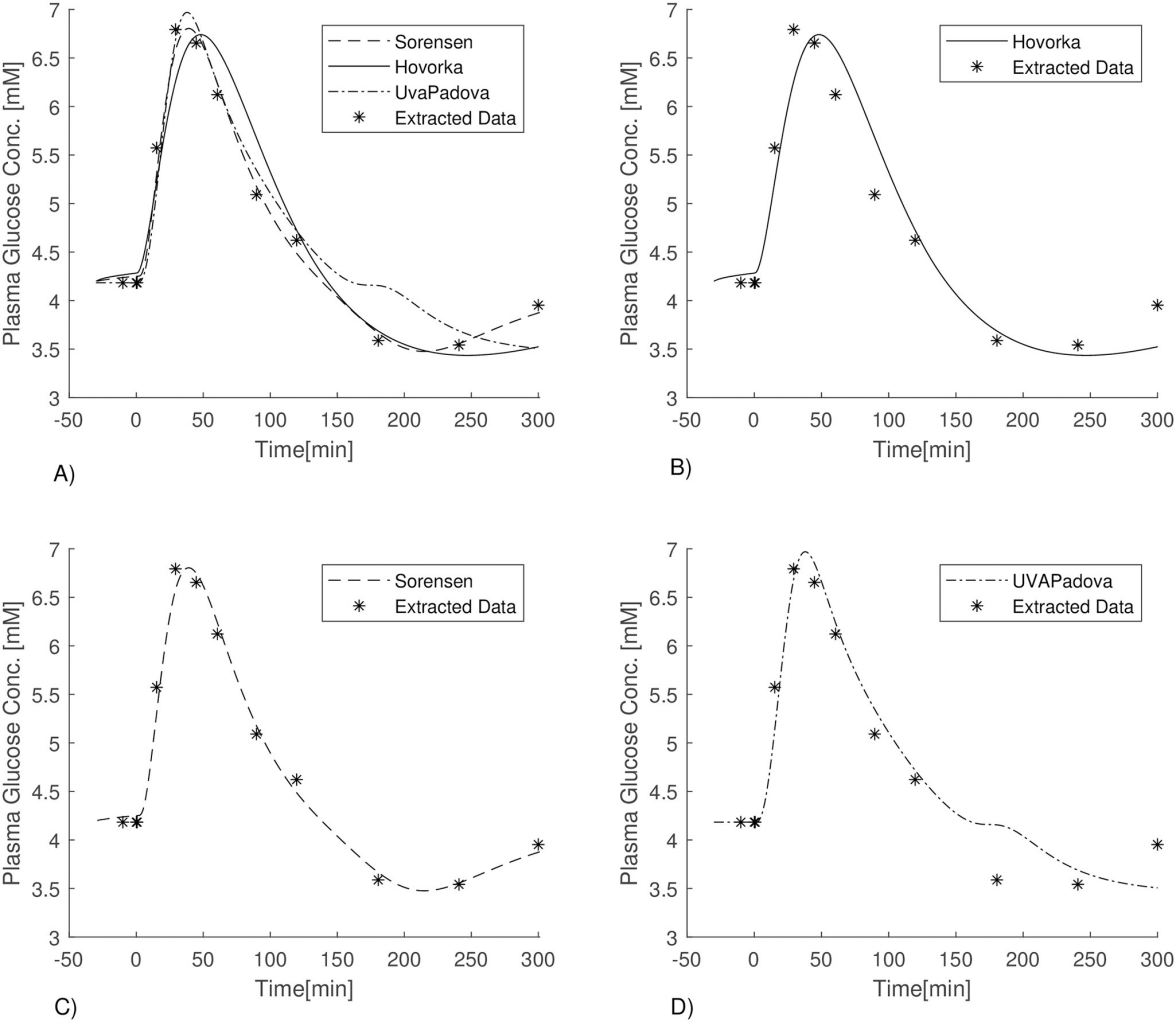
A)Blood glucose concentrations of Sorensen (dashed line), Hovorka (solid line), UVAPadova (dashed dotted line) models with data points from Sorensen PhD thesis (asterisks); B) Hovorka (solid line) vs data points (asterisks); C) Sorensen (dashed line) vs data points (asterisks); D) UVAPadova (dashed dotted line) vs data points (asterisks).

The list of the estimated parameters for the three models, together with their *before* and *after* estimation process values, are reported in Table 6. The last two columns of the table report the Standard Deviations (SDs) and the Coefficients of Variation (CVs) of the estimated parameters. Since the obtained estimate of the parameter *k*_*b*3_ was essentially zero, the parameter was set to 0 and no variability for it was computed. CVs larger than 100% are not reported and they are identified only as being >100%. This happened for all the free parameters of the UVAPadova model. Fig 6 shows the performance of the three formulations.

**Table 6:** Before vs after fitting.

Sorensen	Before	After	SD	CV
*τ*_*S*_(*min*)	55	39.538	1.898	4.78%
*β*_0*PGU*_(#)*	7.03	3.387	0.387	11.41%
*β*_1*PGU*_(#)*	6.52	2.608	0.402	15.42%
**Hovorka**				
$k_{b1}\left(\frac{L}{min^{2}mU}\right)$	3.07E-05	1.571E-04	2.875e-05	18.30%
$k_{b3}\left(\frac{L}{minmU}\right)$	1.56E-03	5.341E-17	/	/
**UVAPadova**				
$k_{1}\left(\frac{1}{min}\right)$	0.042	0.095	0.755	>100%
$k_{2}\left(\frac{1}{min}\right)$	0.071	0.016	0.155	>100%
$k_{p3}\left(\frac{mg}{kgmin}/\frac{pmol}{kg}\right)$	0.014	0.015	0.257	>100%
$\xi (\frac{mg}{kgmin}/\frac{ng}{L})$	0.013	0.011	3.091	>100%
$k_{i}\left(\frac{1}{min}\right)$	0.0066	0.0080	11.461	>100%
$k_{H}\left(\frac{1}{min}\right)$	0.009	0.012	10.313	>100%
$V_{mxB}\left(\frac{mg}{kgmin}/\frac{pmol}{L}\right)$	0.051	0.040	24.735	>100%
$K_{m0}\left(\frac{mg}{kg}\right)$	466.21	0.002	0.045	>100%
$p_{2u}\left(\frac{1}{min}\right)$	0.084	0.050	29.79	>100%

*: $M_{PGU}^{I}=\beta_{0PGU}+\beta_{1PGU}tanh\left[\beta_{2PGU}\left(I_{PI}^{N}-\beta_{3PGU}\right)\right]$

For the Sorensen and Hovorka models, the parameters left free to vary are those related to the external insulin input (*γ*_*SCIin*1_ and *u*_1_, respectively), to the insulin sensitivity mechanism (some parameters in the $M_{PGU}^{I}$ function for the Sorensen model and parameters *k*_*b*1_ and *k*_*b*3_ for the Hovorka model) and to the transfer rates that appear in the subcutaneous insulin compartments (leaving parameter *τ*_*S*_ in Eq [177] to vary only for the Sorensen formulation).

The UVAPadova parameters involved in the fitting procedure are those that appear in the external insulin input (*u*_*scBolo*_), in the representation of insulin sensitivity (*k*_*p*3_, *V*_*mxB*_ and *K*_*m*0_), in the delayed effect of the insulin (*k*_*i*_ and *p*_2*u*_), in the mechanisms of glucose transport between the plasma and tissue compartments (*k*_1_ and *k*_2_) and in the effect of glucagon on glucose production (*ξ* and *k*_*H*_).

While for the Sorensen and Hovorka model it was necessary to optimize the values of four and three parameters respectively, for the UVAPadova model it was necessary to leave ten parameters free to vary to obtain a good fit of the model predictions to data. The greater number of parameters to be estimated for the UVA/Padova formulation may be due to its great complexity. While it is true that the Sorensen model includes the largest number of equations, it should also to be noted that all parameters are set to values compatible with normal physiology. The parameters of the Hovorka and UVA/Padova models are instead indicated for a diabetic individual, but the more compact formulation of the Hovorka model requires fewer modifications to obtain a good fit.

The estimated boluses of insulin required by the three model formulations are: 56387.7 pmol (8886.6 SD, 15.76% CV) (approximately 8 IU), 2361 mU (402.19 SD, 17.03% CV) (approximately 2.3 IU) and 849 pmol/kg (2.508e+05, >100% CV) (approximately 8 IU) for the Sorensen, Hovorka and UVAPadova model respectively. While a similar amount of insulin is necessary for the Sorensen and UVAPadova formulations, the Hovorka model estimated a value four times lower than that estimated by the other two.

Fig 6 shows the glycemia time-course predicted by the three models after the fitting process. As expected, the Sorensen model produces the best fit of predictions to observed concentrations. The Hovorka and UVAPadova models seem to be insufficient to represent the final part of the experiment: the Sorensen model is able to predict the rebound observed around the minute 200, where after a decrease below the basal conditions, there is a recovery towards the baseline.

#### Subsequent three OGTTs in one day

**Fig 7:**
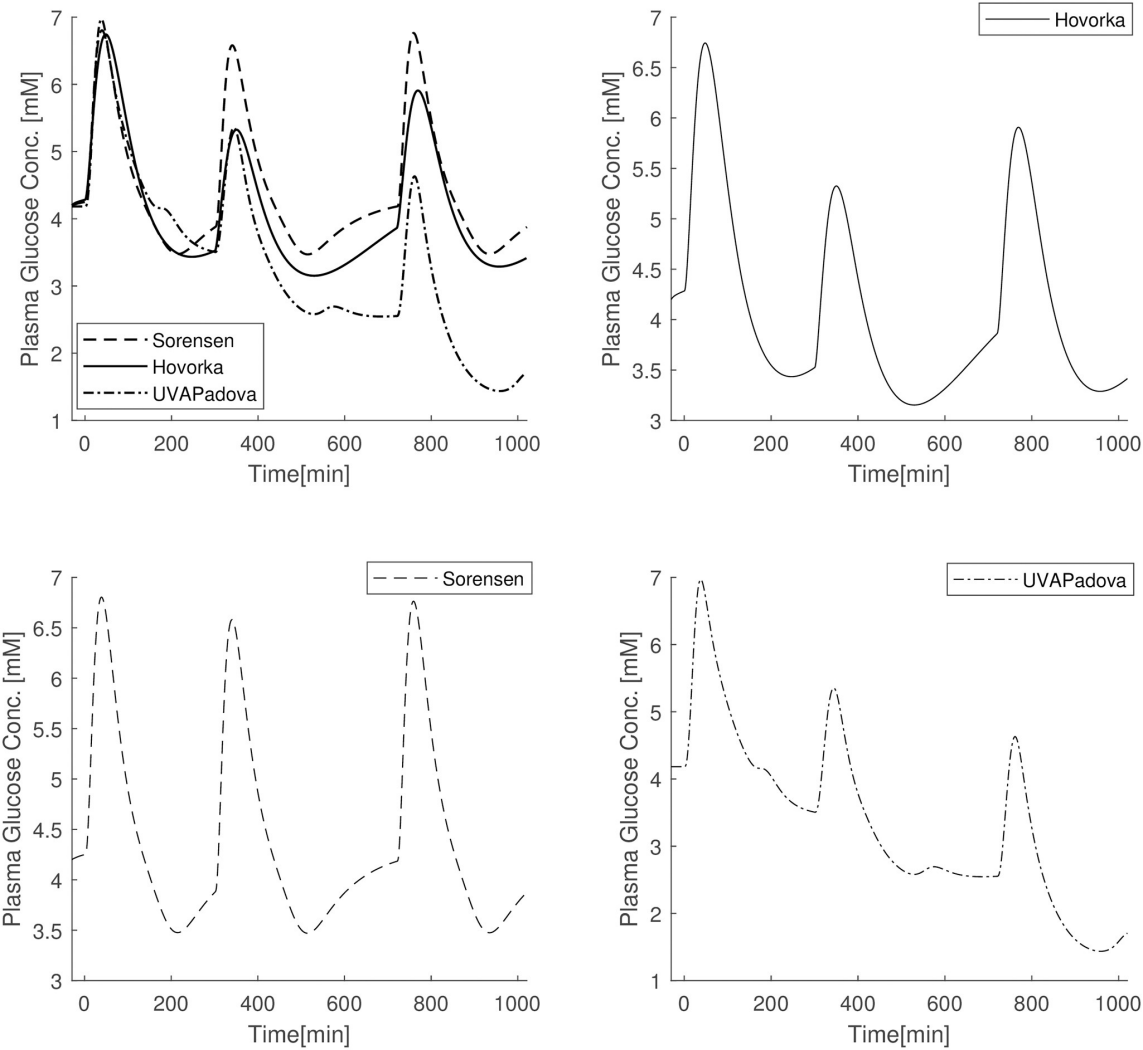
A)Blood glucose concentrations of Sorensen (dashed line), Hovorka (solid line), UVAPadova (dashed dotted line) models after three OGTT in conjunction with three insulin bolus; B) Hovorka (solid line); C) Sorensen (dashed line); UVAPadova (dashed dotted line).

Fig 7 shows the results obtained with three subsequent OGTTs in one day (at 7:00, 12:00 and 19:00) with parameter values set to the estimates obtained by adapting the models to the OGTT data in Fig 6. The Sorensen model appears to be the only one capable of reproducing exactly the same glycemic pattern in the three sub-experiments, with a return to pre-bolus conditions after each OGTT. The UVAPadova formulation is the one for which subsequent OGTTs bring glucose concentrations to lower and lower values (until they fall below a 2mM glycaemia). This feature could be overcome by adopting decreasing values for the parameters *V*_*mxB*_ and *k*_*p*3_, expressing peripheral and central insulin sensitivities respectively, as observed in normal individuals [30]. However, Hinshaw [30] demonstrated that there was no evidence of differences between breakfast and dinner in terms of glucose disappearance in Type 1 diabetic subjects. In the present simulated experiment the values of the two aforesaid parameters were kept constant during the day at the values obtained in the fitting procedure. The Hovorka model predicts glucose concentrations lower than those observed in the first sub-experiment both in the second and third OGTT, nevertheless never producing concentrations below 3mM.

## Discussion

Much work has been done within the scientific community, and is still being done, on the study of appropriate models of the glucose/insulin system, aimed at supporting the development of algorithms for controlled and automatic administration of insulin (the “artificial pancreas”). These models must be able to correctly describe the relevant physiology and need to be identified on each single individual: the ability of a model to provide reliable predictions of the glucose and insulin time courses allows the development of robust control algorithms for automatic glucose control in the management of T1DM patients. Among the models present in the literature, the Sorensen model [20], the Hovorka model [17–19] and the UVAPadova model [22, 24–27] are most frequently used to represent virtual patients to this end. The Sorensen model appears to be, among these three, the most complete and detailed in terms of physiological description and parameter values, with its 22 nonlinear differential equations and 135 parameters. Conversely, the UVAPadova model is the most recent and, judging from the number of publications citing it, is the most frequently used, also because its 2013 [23] version was approved by the FDA.

One limit in using this model, however, lies in the difficulty in deciding the values of its many parameters (about 100). In fact, although the mathematical description of the model appears complete from the aggregated publications describing it, the values of several of its parameters are not published, and this prevents the use of the model by potential users. From a usability point of view, the Hovorka model is the simplest, with few model parameters, and therefore easier use for simulation purposes. With the aim of making them available to the interested scientific community, this work provides a complete description of all three models, together with the values of all of the respective parameters.

The present work compares the three models in terms of their performance when simulating the response of a T1DM individual to different glucose stimuli under different types of insulin administration. The comparison among the three models was performed by implementing four types of *in-silico* experiments.

- The Sorensen [20] model can be defined as a maximal model, as it describes the relationship and interactions between the most important actors of glucose metabolism (glucose, insulin and glucagon) for both a normal individual and a diabetic. This means that, compared to the other two, it provides a description of the role of the pancreas, so as to allow not only the simulation of a Type 1 diabetic patient but also of a Type 2 diabetic subject, as well as a normal individual, for which insulin secretion is still maintained or fully guaranteed, respectively. As mentioned above, the model is well documented with regards to the description of both equations and parameters. It does not provide for a mathematical formalization of the gastrointestinal tract and subcutaneous compartments of insulin, but these two drawbacks can be easily overcome by recovering the missing parts from other model formulations.
- The UVAPadova 2018 [24] model is a maximal model that describes the relationship among glucose, insulin and glucagon only for patients with Type 1 diabetes. Compared to the Sorensen model, the UVAPadova model includes a description of gastric emptying and glucose absorption and offers the possibility of considering different types of insulin and glucagon administration routes (subcutaneous, intradermal, inhaled). Most of the model equations are well documented, but some of these are described only in qualitative terms (as for the *k*_*p*1_ and *k*_*ir*_ functions in Eq [119] and in Eq [123] respectively): these time-varying parameters were therefore kept constant throughout the simulations. The most important flaw however lies in the lack of values for some parameters. Table 4 reports the values of the parameters used in the in-silico experiments, column *Ref*; some of them have been found in the literature and the sources are provided (listed with numbers), some have been determined (indicated with the letter *D*), some have been set at known or reasonable values (denoted by the letter *F*), the rest of the unknown parameters have been calibrated (and are indicated with the letter *C*). Therefore, the model, on the basis of what is reported in literature, is not of immediate implementation and use. The present work provides the scientific community with the most complete description having appeared so far of this model’s equations and parameters, gathering all the information available from the several sources in the literature [22, 24–27].
- The Hovorka model [18] is the simplest among the three analysed models; it describes the relationship between glucose and insulin in subjects with in Type 1 diabetes: therefore, like the UVAPadova model, it does not present a description of the secretion and the release of endogenous insulin. While simpler than the other two, this model does provide a clear formalization of gastro-intestinal absorption after oral administration of glucose, and it includes subcutaneous compartments for the representation of external insulin administration. The Hovorka model appears to be easy to understand, sraightforward to implement, and well documented [17–19].

The Sorensen model, when used for a diabetic individual, is insufficient to adequately describe the response to any type of experiment if no adjustment is made in terms of parameter values. This is due to the fact that, since people with Type 1 diabetes are at risk of severe hyperglycaemia when undergoing perturbation experiments, no perturbation data are available from the literature, so for this Author it was not possible to derive parameter values under these altered conditions. Sorensen therefore adopts the same formalization and quantification used in normal individuals to approximately describe the physiological behaviour of a diabetic subject, apart from the exclusion of the representation of insulin secretion. Fig 1 highlights the behaviour of the Sorensen model, clearly different compared with the other two. The Hovorka and UVAPadova models instead, show similar time courses, reaching the same maximum value with a slight difference in the return to basal conditions. After making the appropriate modifications to the Sorensen model, as described in the “Methods” section, the resulting predictions resemble those obtained with the other two formulations (see Fig 2), although a faster recovery towards the basal values is observed. It is however clear that, in the absence of actual observation data on diabetic subjects, it is not possible to state which of the three models comes closer to describing the correct physiology. Fig 3, mimicking an OGTT without insulin bolus, shows similar trends for Sorensen and UVAPadova, with similar maximum values reached. The Hovorka model predicts higher glucose concentrations and a slower return to baseline conditions. The higher concentrations could be due both to higher than expected endogenous glucose production and to lower tissue glucose uptake in the absence of an insulin bolus.

In Fig 4, where the IVGTT experiment is simulated in conjunction with an insulin bolus, the Hovorka and UVAPadova models show a similar time course, while the predictions of the Sorensen model appear to be qualitatively different. Although no observations on actual patients are available to demonstrate the plausibility of the three models, the predictions obtained with the Sorensen model seem to be more in line with the expected response of a diabetic subject since exhibit a slower return towards pre-experiment values.

In the IVGTT experiment without insulin bolus administration (Fig 4) UVAPadova and Hovorka are quite in agreement, while Sorensen shows a glucose trend more consistent with an insulin-resistant profile. These results have been obtained by modifying some model parameters of the Sorensen formulation, in particular those relating to the description of both central and peripheral insulin sensitivity (Table 5). It is likely that,by changing the values of other parameters, a greater similarity of the predictions of the three models may be obtained. However, in the absence of clear evidence pointing to the better performance of one model with respect to the others, introducing changes in the parameter values chosen by the Author after a thorough literature search is not advisable unless supported by physiological justifications.

The adaptation of the three models to real OGTT data of a normal individual emphasizes the ability of all three models to adapt to real glycemic trends. Even if the setting in which the comparison is made is not optimal (observations are made on a normal individual and not on a diabetic patient), this procedure allows us to investigate the ability of the models to adapt to real data, leaving some parameters free to vary and estimating the administered insulin dose necessary to reproduce the glucose observations. A “good” model should be able to elucidate the relationship between glucose and insulin and should be able to predict a recovery to baseline conditions, with a time course as close as possible to that of a normal subject, with a reasonable amount of insulin. This aspect is important when these models are used in model-based control algorithms, so that the ability of a model to adapt to real observations becomes an essential feature and deviations from what is observed emphasize important physiological deficiencies.

Fig 6 shows a very good performance of all three models in adapting to the data, but we can see that the Sorensen model is the only one able to predict the recovery phase with a rebound after a hypo-glycaemic period produced by the administration of insulin, reaching the last available data point. This could be due to the fact that the parameter values used for the Sorensen model were those originally adapted to the physiology of a normal individual. Surprisingly, while the Hovorka model tries to predict a recovery phase, which will indeed occur at a later time, the UVA/Padova model appears to stabilize at a lower blood glucose levels despite a greater number of free model parameters (ten for UVa-Padova, four for Sorensen and three for Hovorka). The estimated amount of insulin administered as a bolus, needed to obtain the predictions of Fig 6 are 2.3 IU, 8.5 IU and 8 IU for the Hovorka, UVAPadova and Sorensen formulations respectively. The much lower value observed for the Hovorka model could depend on the estimated values of the other free parameters: insulin sensitivity, explicitly represented in the Hovorka model by the parameter *k*_*b*1_, is estimated in fact at about 1.6 × 10^−4^, and if insulin sensitivity is high, then necessary amount of insulin to be delivered decreases. In this context however it should be noted that a lower *k*_*b*3_ (roughly representing central peripheral insulin) determines greater production of endogenous glucose, which on one hand compensates for the augmented sensitivity to peripheral insulin, while on the other causes final recovery to attain a higher blood glucose concentration. Concurrently with a large bolus of insulin obtained for the Sorensen model, the parameters of the $M_{PGU}^{I}$ function (representing insulin sensitivity) all decreased by about a factor of two. For the UVAPadova model an external insulin input (*u*_*scBolo*_) of approximately 8 IU is required. A much lower *K*_*m*0_ parameter (approximately zero) in Eq (123) is consistent with much improved insulin sensitivity, accelerating the effect of insulin on glucose utilization.

### Conclusion

The three models seem to reproduce all the simulated experimental situations quite well without obvious divergences. The Sorensen model produces predictions similar to those of the other two models once some parameter values are modified, suggesting that, with suitable adaptation, this model could be used to also represent the physiology of diabetic subjects. An updated formulation including both the gastrointestinal tract and the subcutaneous insulin deposit compartment should be used in this case.

With the information available at the present time, the final choice about which model to use lies in the confidence that the experimenter places on how plausibly the mathematics represents the underlying physiology, as well as on the simplicity, robustness and versatility of the formulation. A more complex model with a large number of parameters might in principle fit better with observations, but the complexity of a model not only makes identification statistically harder, but it suffers from possible over-fitting and consequently fragile, unreliable forecasts. From this point of view, the Sorensen model is the one with the greatest number of parameters (adding the fixed, determined and free ones). However, since they are well documented and an in-depth bibliographic research has been carried out by the Author, most of the parameters are set to known values, so as to require the estimation of a lower number of free parameters than the UVAPadova model, which in any case is poorly documented. Conversely, Hovorka’s model is the simplest, and still fits sufficiently well the observed data. We notice however that the Sorensen model is the only one capable of predicting the rebound phase in the OGTT experiments, where the other two models fail.

From purely *in silico* experiments it is not possible to draw definitive conclusions on which model is physiologically more credible. The next logical step in the evaluation of these and possibly other maximal models of the glucose-insulin system would be to compare their predictions against actual observational data, obtained with different experimental set-ups in patients with a range of normal and diabetes conditions.

## Appendix

### The Sorensen Model

**Mass Balance—Glucose**
**BRAIN**
$$\begin{array}{c} V_{BV}^{G}\frac{dG_{BV}}{dt}=Q_{B}^{G}\left(G_{H}-G_{BV}\right)-\frac{V_{BI}}{T_{B}}\left(G_{BV}-G_{BI}\right) \end{array}$$ (8)
$$\begin{array}{c} V_{BI}\frac{dG_{BI}}{dt}=\frac{V_{BI}}{T_{B}}\left(G_{BV}-G_{BI}\right)-r_{BGU} \end{array}$$ (9)
**HEART AND LUNGS**
$$\begin{array}{c} \begin{array}{cc} V_{H}^{G}\frac{dG_{H}}{dt} & =Q_{B}^{G}G_{BV}+Q_{L}^{G}G_{L}+Q_{K}^{G}G_{K}+\\ & +Q_{P}^{G}G_{PV}-Q_{H}^{G}G_{H}-r_{RBCU} \end{array} \end{array}$$ (10)
**GUT**
$$\begin{array}{c} V_{J}^{G}\frac{dG_{J}}{dt}=Q_{J}^{G}\left(G_{H}-G_{J}\right)-r_{JGU} \end{array}$$ (11)
**LIVER**
$$\begin{array}{c} V_{L}^{G}\frac{dG_{L}}{dt}=Q_{A}^{G}G_{H}+Q_{J}^{G}G_{J}-Q_{L}^{G}G_{L}+r_{HGP}-r_{HGU} \end{array}$$ (12)
**KIDNEY**
$$\begin{array}{c} V_{K}^{G}\frac{dG_{K}}{dt}=Q_{K}^{G}\left(G_{H}-G_{K}\right)-r_{KGE} \end{array}$$ (13)
**PERIPHERY**
$$\begin{array}{c} V_{PV}^{G}\frac{dG_{PV}}{dt}=Q_{P}^{G}\left(G_{H}-G_{PV}\right)-\frac{V_{PI}}{T_{P}^{G}}\left(G_{PV}-G_{PI}\right) \end{array}$$ (14)
$$\begin{array}{c} V_{PI}\frac{dG_{PI}}{dt}=\frac{V_{PI}}{T_{P}^{G}}\left(G_{PV}-G_{PI}\right)-r_{PGU} \end{array}$$ (15)
**Metabolic Source and Sinks—Glucose**
$$\begin{array}{c} r_{BGU}=70\frac{mg}{min}\;\left[constant\right] \end{array}$$ (16)
$$\begin{array}{c} r_{RBCU}=10\frac{mg}{min}\;\left[constant\right] \end{array}$$ (17)
$$\begin{array}{c} r_{JGU}=20\frac{mg}{min}\;\left[constant\right] \end{array}$$ (18)
$$\begin{array}{c} r_{PGU}=M_{PGU}^{I}M_{PGU}^{G}r_{PGU}^{B} \end{array}$$ (19)
$$\begin{array}{c} r_{PGU}^{B}=35\frac{mg}{min} \end{array}$$ (20)
$$\begin{array}{c} M_{PGU}^{I}=7.03+6.52tanh\left[0.338\left(I_{PI}^{N}-5.82\right)\right] \end{array}$$ (21)
$$\begin{array}{c} M_{PGU}^{G}=G_{PI}^{N} \end{array}$$ (22)
$$\begin{array}{c} r_{HGP}=M_{HGP}^{I}M_{HGP}^{\Gamma }M_{HGP}^{G}r_{HGP}^{B} \end{array}$$ (23)
$$\begin{array}{c} r_{HGP}^{B}=155\frac{mg}{min} \end{array}$$ (24)
$$\begin{array}{c} \frac{dM_{HGP}^{I}}{dt}=\frac{1}{\tau_{I}}\left[M_{HGP}^{I_{\infty }}-M_{HGP}^{I}\right] \end{array}$$ (25)
$$\begin{array}{c} \tau_{I}=25min \end{array}$$ (26)
$$\begin{array}{c} M_{HGP}^{I_{\infty }}=1.21-1.14tanh\left[1.66\left(I_{L}^{N}-0.89\right)\right] \end{array}$$ (27)
$$\begin{array}{c} M_{HGP}^{\Gamma }=M_{HGP}^{\Gamma_{0}}-f_{2} \end{array}$$ (28)
$$\begin{array}{c} M_{HGP}^{\Gamma_{0}}=2.7tanh\left[0.39\Gamma^{N}\right] \end{array}$$ (29)
$$\begin{array}{c} \frac{df_{2}}{dt}=\frac{1}{\tau_{\Gamma }}\left(\frac{M_{HGP}^{\Gamma_{0}}-1}{2}-f_{2}\right) \end{array}$$ (30)
$$\begin{array}{c} \tau_{\Gamma }=65min \end{array}$$ (31)
$$\begin{array}{c} M_{HGP}^{G}=1.42-1.41tanh\left[0.62\left(G_{L}^{N}-0.497\right)\right] \end{array}$$ (32)
$$\begin{array}{c} r_{HGU}=M_{HGU}^{I}M_{HGU}^{G}r_{HGU}^{B} \end{array}$$ (33)
$$\begin{array}{c} r_{HGU}^{B}=20\frac{mg}{min} \end{array}$$ (34)
$$\begin{array}{c} \frac{dM_{HGU}^{I}}{dt}=\frac{1}{\tau_{I}}\left[M_{HGU}^{I_{\infty }}-M_{HGU}^{I}\right] \end{array}$$ (35)
$$\begin{array}{c} M_{HGU}^{I_{\infty }}=2tanh\left[0.55I_{L}^{N}\right] \end{array}$$ (36)
$$\begin{array}{c} M_{HGU}^{G}=5.66+5.66tanh\left[2.44\left(G_{L}^{N}-1.48\right)\right] \end{array}$$ (37)
$$\begin{array}{c} r_{KGE}=\{\begin{array}{cc} 71+71tanh[0.011\left(G_{K}-460\right)] & 0<G_{K}<460\frac{mg}{min}\\ -330+0.872G_{K} & G_{K}\geq 460\frac{mg}{min} \end{array} \end{array}$$ (38)
**Mass Balance—Insulin**
**BRAIN**
$$\begin{array}{c} V_{B}^{I}\frac{dI_{B}}{dt}=Q_{B}^{I}\left(I_{H}-I_{B}\right) \end{array}$$ (39)
**HEART AND LUNGS**
$$\begin{array}{c} \begin{array}{cc} V_{H}^{I}\frac{dI_{H}}{dt} & =Q_{B}^{I}I_{B}+Q_{L}^{I}I_{L}+Q_{K}^{I}I_{K}+\\ & +Q_{P}^{I}I_{PV}-Q_{H}^{I}I_{H}+\gamma_{IVI} \end{array} \end{array}$$ (40)
**GUT**
$$\begin{array}{c} V_{J}^{I}\frac{dI_{J}}{dt}=Q_{J}^{I}\left(I_{H}-I_{J}\right) \end{array}$$ (41)
**LIVER**
$$\begin{array}{c} V_{L}^{I}\frac{dI_{L}}{dt}=Q_{A}^{I}I_{H}+Q_{J}^{I}I_{J}-Q_{L}^{I}I_{L}+r_{PIR}-r_{LIC} \end{array}$$ (42)
**KIDNEY**
$$\begin{array}{c} V_{K}^{I}\frac{dI_{K}}{dt}=Q_{K}^{I}\left(I_{H}-I_{K}\right)-r_{KIC} \end{array}$$ (43)
**PERIPHERY**
$$\begin{array}{c} V_{PV}^{I}\frac{dI_{PV}}{dt}=Q_{P}^{I}\left(I_{H}-I_{PV}\right)-\frac{V_{PI}}{T_{PI}^{I}}\left(I_{PV}-I_{PI}\right) \end{array}$$ (44)
$$\begin{array}{c} V_{PI}\frac{dI_{PI}}{dt}=\frac{V_{PI}}{T_{P}^{I}}\left(I_{PV}-I_{PI}\right)-r_{PIC} \end{array}$$ (45)
**Metabolic Source and Sinks—Insulin**
$$\begin{array}{c} r_{LIC}=F_{LIC}\left[Q_{A}^{I}I_{H}+Q_{J}^{I}I_{J}+r_{PIR}\right] \end{array}$$ (46)
$$\begin{array}{c} F_{LIC}=0.40 \end{array}$$ (47)
$$\begin{array}{c} r_{KIC}=F_{KIC}\left[Q_{K}^{I}I_{H}\right] \end{array}$$ (48)
$$\begin{array}{c} F_{KIC}=0.30 \end{array}$$ (49)
$$\begin{array}{c} r_{PIC}=\frac{I_{PI}}{\left[\left(\frac{1-F_{PIC}}{F_{PIC}}\right)\left(\frac{1}{Q_{P}^{I}}\right)-\frac{T_{P}^{I}}{V_{PI}}\right]} \end{array}$$ (50)
$$\begin{array}{c} F_{PIC}=0.15 \end{array}$$ (51)
$$\begin{array}{c} r_{PIR}=\frac{S(G_{H})}{S(G_{H}^{B})}r_{PIR}^{B} \end{array}$$ (52)
$$\begin{array}{c} \frac{dP}{dt}=\alpha \left[P_{\infty }-P\right] \end{array}$$ (53)
$$\begin{array}{c} \frac{dI}{dt}=\beta \left[X-I\right] \end{array}$$ (54)
$$\begin{array}{c} \frac{dQ}{dt}=K\left(Q_{0}-Q\right)+\gamma P-S \end{array}$$ (55)
$$\begin{array}{c} S=[M_{1}Y+M_{2}{\left(X-I\right)}^{0^{+}}]Q \end{array}$$ (56)
$$\begin{array}{c} X=\frac{{\left(G_{H}\right)}^{3.27}}{{\left(132\right)}^{3.27}+5.93{\left(G_{H}\right)}^{3.02}} \end{array}$$ (57)
$$\begin{array}{c} P_{\infty }=Y={\left(X\right)}^{1.11} \end{array}$$ (58)
**Mass Balance—Glucagon**
$$\begin{array}{c} V^{\Gamma }\frac{d\Gamma }{dt}=r_{P\Gamma R}-r_{P\Gamma C} \end{array}$$ (59)
**Metabolic Source and Sinks—Glucagon**
$$\begin{array}{c} r_{P\Gamma R}=r_{M\Gamma C}\Gamma \end{array}$$ (60)
$$\begin{array}{c} r_{M\Gamma C}=9.10\frac{ml}{min} \end{array}$$ (61)
$$\begin{array}{c} r_{P\Gamma R}=M_{P\Gamma R}^{G}M_{P\Gamma R}^{I}r_{P\Gamma R}^{B} \end{array}$$ (62)
$$\begin{array}{c} M_{P\Gamma R}^{G}=2.93-2.10tanh\left[4.18\left(G_{H}^{N}-0.61\right)\right] \end{array}$$ (63)
$$\begin{array}{c} M_{P\Gamma R}^{I}=1.31-0.61tanh\left[1.06\left(I_{H}^{N}-0.47\right)\right] \end{array}$$ (64)
**Parameter values** ^1^
**Glucose** $\begin{array}{ccc} V_{BV}^{G}=3.5dL & Q_{B}^{G}=5.9\frac{dL}{min} & T_{B}=2.1min\\ V_{BI}=4.5dL & Q_{H}^{G}=43.7\frac{dL}{min} & T_{P}^{G}=5.0min\\ V_{H}^{G}=13.8dL & Q_{A}^{G}=2.5\frac{dL}{min} & \\ V_{L}^{G}=25.1dL & Q_{L}^{G}=12.6\frac{dL}{min} & \\ V_{J}^{G}=11.2dL & Q_{G}^{G}=10.1\frac{dL}{min} & \\ V_{K}^{G}=6.6dL & Q_{K}^{G}=10.1\frac{dL}{min} & \\ V_{PV}^{G}=10.4dL & Q_{P}^{G}V=15.1\frac{dL}{min} & \\ V_{PI}=67.4dL & & \end{array}$

**Insulin** $\begin{array}{ccc} V_{B}^{I}=0.26L & Q_{B}^{I}=0.45\frac{L}{min} & T_{P}^{I}=20min\\ V_{H}^{I}=0.99L & Q_{H}^{I}=3.12\frac{L}{min} & \\ V_{G}^{I}=0.94L & Q_{A}^{I}=0.18\frac{L}{min} & \\ V_{L}^{I}=1.14L & Q_{K}^{I}=0.72\frac{L}{min} & \\ V_{K}^{I}=0.51L & Q_{P}^{I}=1.05\frac{L}{min} & \\ V_{PV}^{I}=0.74L & Q_{J}^{I}=0.72\frac{L}{min} & \\ V_{PI}^{I}=6.74L & Q_{L}^{I}=0.90\frac{L}{min} & \\ M_{1}=0.00747min^{-1} & M_{2}=0.0958min^{-1} & Q_{0}=6.33U\\ \alpha =0.0482min^{-1} & \beta =0.931min^{-1} & K=0.575\frac{U}{min} \end{array}$

  **Glucagon***V*^Γ^ = 11310*ml*
  1: Sorensen PhD thesis [20]

#### Determined parameters

$$\begin{array}{c} S_{10}=\gamma_{SCI0}\tau_{S} \end{array}$$ (65)

$$\begin{array}{c} S_{20}=S_{10} \end{array}$$ (66)

$$\begin{array}{c} I_{H0}=\frac{\frac{S_{20}}{\tau_{S}}+\gamma_{IVI0}}{Q_{H}^{I}-Q_{L}^{I}\left(1-F_{LIC}\right)-Q_{K}^{I}\left(1-F_{KIC}\right)-Q_{P}^{I}\left(1-F_{PIC}\right)-Q_{B}^{I}} \end{array}$$ (67)

$$\begin{array}{c} I_{PV0}=I_{H0}\left(1-F_{PIC}\right) \end{array}$$ (68)

$$\begin{array}{c} I_{K0}=I_{H0}\left(1-F_{KIC}\right) \end{array}$$ (69)

$$\begin{array}{c} I_{B0}=I_{H0} \end{array}$$ (70)

$$\begin{array}{c} I_{J0}=I_{H0} \end{array}$$ (71)

$$\begin{array}{c} I_{L0}=I_{H0}\left(1-F_{LIC}\right) \end{array}$$ (72)

$$\begin{array}{c} I_{L0}^{N}=\frac{I_{L0}}{I_{L}^{B}} \end{array}$$ (73)

$$\begin{array}{c} I_{PI0}^{N}=\frac{I_{PI0}}{I_{PI}^{B}} \end{array}$$ (74)

$$\begin{array}{c} I_{H0}^{N}=\frac{I_{H0}}{I_{H}^{B}} \end{array}$$ (75)

$$\begin{array}{c} r_{PIC0}=\frac{I_{PI0}}{\frac{1-F_{PIC}}{F_{PIC}}\frac{1}{Q_{IP}}-\frac{T_{P}^{I}}{V_{PI}}} \end{array}$$ (76)

$$\begin{array}{c} M_{PGU0}=\beta_{0PGU}+\beta_{1PGU}tanh\left[\beta_{2PGU}\left(I_{PI0}^{N}-\beta_{3PGU}\right)\right] \end{array}$$ (77)

$$\begin{array}{c} G_{PV0}=\frac{G_{H0}}{1+\frac{V_{PI}M_{PGU0}^{I}r_{PGU}^{B}}{Q_{P}^{G}V_{PI}G_{PI}^{B}+T_{P}^{G}Q_{P}^{G}M_{PGU0}^{I}r_{PGU}^{B}}} \end{array}$$ (78)

$$\begin{array}{c} G_{BV0}=G_{H0}-\frac{r_{BGU}}{Q_{B}^{G}} \end{array}$$ (79)

$$\begin{array}{c} G_{J0}=G_{H0}-\frac{r_{JGU}}{Q_{J}^{G}} \end{array}$$ (80)

$$\begin{array}{c} G_{BI0}=G_{BV0}-\frac{r_{BGU}T_{B}}{V_{BI}} \end{array}$$ (81)

$$\begin{array}{c} G_{PI0}=\frac{G_{PV0}}{1+\frac{M_{PGU0}^{G}r_{PGU}^{B}T_{P}^{G}}{V_{PI}G_{PI}^{B}}} \end{array}$$ (82)

$$\begin{array}{c} G_{H0}^{N}=\frac{G_{H0}}{G_{H}^{B}} \end{array}$$ (83)

$$\begin{array}{c} G_{PI0}^{N}=\frac{G_{PI0}}{G_{PI}^{B}} \end{array}$$ (84)

$$\begin{array}{c} G_{L0}^{N}=\frac{G_{L0}}{G_{L}^{B}} \end{array}$$ (85)

$$\begin{array}{c} M_{HGP0}^{I}=\beta_{2HGP}+\beta_{3HGP}tanh\left[\beta_{4HGP}\left(I_{L0}^{N}-\beta_{5HGP}\right)\right] \end{array}$$ (86)

$$\begin{array}{c} M_{HGPinf}^{I}=M_{HGP0}^{I} \end{array}$$ (87)

$$\begin{array}{c} M_{HGP0}^{G}=\beta_{6HGP}+\beta_{7HGP}tanh\left[\beta_{8HGP}\left(G_{L0}^{N}-\beta_{9HGP}\right)\right] \end{array}$$ (88)

$$\begin{array}{c} M_{HGU0}^{I}=\beta_{0HGU}tanh\left(\beta_{1HGU}I_{L0}^{N}\right) \end{array}$$ (89)

$$\begin{array}{c} M_{HGUinf}^{I}=M_{HGU0}^{I} \end{array}$$ (90)

$$\begin{array}{c} M_{HGU0}^{G}=\beta_{2HGU}+\beta_{3HGU}tanh\left[\beta_{4HGU}\left(G_{L0}^{N}-\beta_{5HGU}\right)\right] \end{array}$$ (91)

$$\begin{array}{ccc} r_{KGE0} & = & \left\{ \begin{array}{cc} \beta_{KGE0}+\beta_{KGE1}\;\tanh[\beta_{KGE2}\;\left(G_{K0}-\beta_{KGE3}\right)] & ,\;0\leq G_{K0}<\beta_{KGE3}\\ -\beta_{KGE4}+\beta_{KGE5}\;G_{K0} & ,\;G_{K0}\geq \beta_{KGE3} \end{array}\right. \end{array}$$ (92)

$$\begin{array}{c} r_{LIC0}=F_{LIC}\left(Q_{A}^{I}I_{H0}+Q_{J}^{I}I_{J0}+r_{BPIR}\right) \end{array}$$ (93)

$$\begin{array}{c} r_{KIC0}=F_{KIC}\left(Q_{K}^{I}I_{H0}\right) \end{array}$$ (94)

$$\begin{array}{c} M_{PCR0}^{G}=\beta_{0PCR}+\beta_{1PCR}tanh\left[\beta_{3PCR}\left(G_{H0}^{N}-\beta_{3PCR}\right)\right] \end{array}$$ (95)

$$\begin{array}{c} M_{PCR0}^{I}=\beta_{4PCR}+\beta_{5PCR}tanh\left[\beta_{6PCR}\left(I_{H0}^{N}-\beta_{7PCR}\right)\right] \end{array}$$ (96)

$$\begin{array}{c} \Gamma_{N0}=M_{P\Gamma R0}^{I}M_{P\Gamma R0}^{G} \end{array}$$ (97)

$$\begin{array}{c} M_{HGP0}^{\Gamma 0}=\beta_{0HGP}tanh\left(\beta_{1HGP}\Gamma_{0}^{N}\right) \end{array}$$ (98)

$$\begin{array}{c} f_{20}=\frac{M_{HGP0}^{\Gamma 0}-1}{2} \end{array}$$ (99)

$$\begin{array}{c} M_{HGP0}^{\Gamma }=\beta_{0HGP}tanh\left(\beta_{1HGP}\Gamma_{0}^{N}\right)-f_{20} \end{array}$$ (100)

$$\begin{array}{c} r_{P\Gamma C0}=\Gamma_{0}^{N}r_{M\Gamma C} \end{array}$$ (101)

$$\begin{array}{c} r_{BP\Gamma R}=\frac{\Gamma_{0}r_{M\Gamma C}}{M_{P\Gamma R0}^{G}M_{P\Gamma R0}^{I}} \end{array}$$ (102)

$$\begin{array}{c} r_{PGU0}=M_{PGU0}^{I}G_{PI0}^{N}r_{BPGU} \end{array}$$ (103)

$$\begin{array}{c} r_{HGP0}=r_{HGP0}M_{HGP0}^{I}M_{HGP0}^{\Gamma }M_{HGP0}^{G} \end{array}$$ (104)

$$\begin{array}{c} r_{HGU0}=r_{HGU0}M_{HGU0}^{I}M_{HGU0}^{G} \end{array}$$ (105)

$$\begin{array}{c} r_{P\Gamma R0}=\Gamma_{0}r_{M\Gamma C} \end{array}$$ (106)

### The UVAPadova Model

**Glucose Model**
$$\begin{array}{c} \begin{array}{cc} \frac{dG_{p}}{dt} & =EGP\left(t\right)+Ra_{meal}\left(t\right)-U_{ii}-E\left(t\right)+\\ & -k_{1}G_{p}\left(t\right)+k_{2}G_{t}\left(t\right) \end{array} \end{array}$$ (107)
$$\begin{array}{c} \frac{dG_{t}}{dt}=-U_{id}\left(t\right)+k_{1}G_{p}\left(t\right)-k_{2}G_{t}\left(t\right) \end{array}$$ (108)
$$\begin{array}{c} G\left(t\right)=\frac{G_{p}\left(t\right)}{V_{G}} \end{array}$$ (109)
**Insulin Model**
$$\begin{array}{c} \frac{dI_{p}}{dt}=-\left(m_{2}+m_{4}\right)I_{p}\left(t\right)+m_{1}I_{l}\left(t\right)+Ra_{I}\left(t\right) \end{array}$$ (110)
$$\begin{array}{c} \frac{dI_{p}}{dt}=-\left(m_{1}+m_{3}\right)I_{l}\left(t\right)+m_{2}I_{p}\left(t\right) \end{array}$$ (111)
$$\begin{array}{c} I\left(t\right)=\frac{I_{p}\left(t\right)}{V_{I}} \end{array}$$ (112)
**Gastrointestinal Model**
$$\begin{array}{c} Q_{sto}\left(t\right)=Q_{sto1}\left(t\right)+Q_{sto2}\left(t\right) \end{array}$$ (113)
$$\begin{array}{c} \frac{dQ_{sto1}}{dt}=-k_{max}Q_{sto1}\left(t\right)+\sum_{i=1}^{N_{p}}Dose\delta \left(t-t_{i}\right) \end{array}$$ (114)
  The equation from UVAPadova S2017 [24] is changed because it is incorrect with respect to the operating conditions.
  - *N*_*p*_ = number of meals (maximum 3)
  - *i* = 1 (Breakfast)
  - *i* = 2 (Lunch)
  - *i* = 3 (Dinner)
$$\begin{array}{c} \frac{dQ_{sto2}}{dt}=-k_{empt}\left(Q_{sto}\right)Q_{sto2}\left(t\right)+k_{max}Q_{sto1}\left(t\right) \end{array}$$ (115)
$$\begin{array}{c} \frac{dQ_{gut}}{dt}=-k_{abs}Q_{gut}\left(t\right)+k_{empt}\left(Q_{sto}\right)Q_{sto2}\left(t\right) \end{array}$$ (116)
$$\begin{array}{c} Ra_{meal}\left(t\right)=\frac{fk_{abs}Q_{gut}\left(t\right)}{BW} \end{array}$$ (117)
$$\begin{array}{c} \begin{array}{cc} k_{empt}\left(Q_{sto}\right) & =k_{min}+\frac{k_{max}-k_{min}}{2}\{tanh[\alpha (Q_{sto}+\\ & -bDose)]-tanh\left[\beta \left(Q_{sto}-cDose\right)\right]+\\ & +2\} \end{array} \end{array}$$ (118)
**EGP Model**
$$\begin{array}{c} EGP\left(t\right)=k_{p1}-k_{p2}G_{p}\left(t\right)-k_{p3}X^{L}\left(t\right)+\xi X^{H}\left(t\right) \end{array}$$ (119)
$$\begin{array}{c} \frac{dX^{L}}{dt}=-k_{i}\left[X^{L}\left(t\right)-I^{{}^{\prime }}\left(t\right)\right] \end{array}$$ (120)
$$\begin{array}{c} \frac{dI^{{}^{\prime }}}{dt}=-k_{i}\left[I^{{}^{\prime }}\left(t\right)-I\left(t\right)\right] \end{array}$$ (121)
$$\begin{array}{c} \frac{dX^{H}}{dt}=-k_{H}X^{H}\left(t\right)+k_{H}max\left[\left(H\left(t\right)-H_{b}\right),0\right] \end{array}$$ (122)
**Glucose Utilization Model**
$$\begin{array}{c} U_{id}\left(t\right)=\frac{k_{ir}\left[V_{m0}+V_{mx}X\left(t\right)\left(1+r_{1}risk\right)\right]G_{t}\left(t\right)}{K_{m0}+G_{t}\left(t\right)} \end{array}$$ (123)
  The equation from UVAPadova S2017 [24] is replaced by that in UVAPadova (2014) [23], because reported with an error.
  $$\begin{array}{c} \frac{dX}{dt}=-p_{2U}X\left(t\right)+p_{2U}\left[I\left(t\right)-I_{b}\right] \end{array}$$ (124)
  $$\begin{array}{c} risk\{\begin{array}{cc} 0 & G\geq G_{b}\\ 10f_{1}^{2} & G_{th}\leq G<G_{b}\\ 10f_{2}^{2} & G<G_{th} \end{array} \end{array}$$ (125)
  $$\begin{array}{c} f_{1}={\left(log\left(G\right)\right)}^{r_{2}}-{\left(log\left(G_{b}\right)\right)}^{r_{2}} \end{array}$$ (126)
**Renal Elimination**
$$\begin{array}{c} E\left(t\right)\{\begin{array}{cc} \left(k_{e1}\left(G_{p}\left(t\right)-k_{e2}\right)\right) & G_{p}\left(t\right)>k_{e2}\\ 0 & G_{p}\left(t\right)\leq k_{e2} \end{array} \end{array}$$ (127)
**Insulin Rate of Appearance Model**
$$\begin{array}{c} Ra_{I}\left(t\right)=Ra_{Isc}\left(t\right)+Ra_{Iih}\left(t\right) \end{array}$$ (128)
**Subcutaneous insulin kinetics**
$$\begin{array}{c} Ra_{Isc}\left(t\right)=k_{a1}I_{sc1}\left(t\right)+k_{a2}I_{sc2}\left(t\right) \end{array}$$ (129)
$$\begin{array}{c} \frac{dI_{sc1}}{dt}=-\left(k_{d}+k_{a1}\right)I_{sc1}\left(t\right)+u_{sc}\left(t-\tau \right) \end{array}$$ (130)
$$\begin{array}{c} \frac{dI_{sc2}}{dt}=k_{d}I_{sc1}\left(t\right)-k_{a2}I_{sc2} \end{array}$$ (131)
**Inhaled insulin kinetics**
$$\begin{array}{c} Ra_{Iih}\left(t\right)=k_{aIih}I_{ih}\left(t\right) \end{array}$$ (132)
$$\begin{array}{c} \frac{dI_{ih}}{dt}=-k_{aIih}I_{ih}\left(t\right)+F_{Iih}u_{ih}\left(t\right) \end{array}$$ (133)
**Subcutaneous glucose kinetics**
$$\begin{array}{c} \frac{dG_{sc}}{dt}=-\frac{1}{T_{s}}G_{sc}\left(t\right)+\frac{1}{T_{s}}G\left(t\right) \end{array}$$ (134)
**Glucagon kinetics and secretion**
$$\begin{array}{c} \frac{dH}{dt}=-nH\left(t\right)+SR_{H}\left(t\right)+Ra_{H}\left(t\right) \end{array}$$ (135)
$$\begin{array}{c} SR_{H}\left(t\right)=SR_{H}^{S}\left(t\right)+SR_{H}^{d}\left(t\right) \end{array}$$ (136)
$$\begin{array}{c} SR_{H}^{S}\left(t\right)=\{\begin{array}{cc} \rho [SR_{H}^{S}\left(t\right)-SR_{H}^{b}] & G\left(t\right)\geq G_{b}\\ \rho [SR_{H}^{S}\left(t\right)-max\left(\sigma \frac{G_{th}-G\left(t\right)}{I(t)+1}+SR_{H}^{b},0\right)] & G\left(t\right)<G_{b} \end{array} \end{array}$$ (137)
$$\begin{array}{c} SR_{H}^{d}\left(t\right)=\delta max\left[-\frac{dG(t)}{dt},0\right] \end{array}$$ (138)
**Subcutaneous Glucagon kinetics**
$$\begin{array}{c} \frac{dH_{sc1}}{dt}=-\left(k_{h1}+k_{h2}\right)H_{sc1}\left(t\right) \end{array}$$ (139)
$$\begin{array}{c} \frac{dH_{sc2}}{dt}=k_{h1}H_{sc1}\left(t\right)-k_{h3}H_{sc2}\left(t\right) \end{array}$$ (140)
**Rate of appearance of the Glucagon**
$$\begin{array}{c} Ra_{H}\left(t\right)=k_{h3}H_{sc2}\left(t\right) \end{array}$$ (141)

#### Determined parameters

$$\begin{array}{c} G_{pb}\left(t\right)=V_{G}G_{b} \end{array}$$ (142)

$$\begin{array}{c} E_{0}=\{\begin{array}{cc} (k_{e1}*\left(G_{pb}-k_{e2}\right) & G_{pb}>k_{e2}\\ 0 & G_{pb}\leq k_{e2} \end{array} \end{array}$$ (143)

$$\begin{array}{c} I_{pb}=\frac{u_{sc0}}{m_{2}+m_{4}-\frac{m_{1}m_{2}}{m_{1}+m_{3}}} \end{array}$$ (144)

$$\begin{array}{c} I_{b}=\frac{I_{pb}}{V_{I}} \end{array}$$ (145)

$$\begin{array}{c} X_{0}^{L}=I_{b} \end{array}$$ (146)

$$\begin{array}{c} k_{p1}=EGP_{0}+k_{p2}G_{pb}+k_{p30}X_{0}^{L} \end{array}$$ (147)

$$\begin{array}{c} G_{tb}=\frac{U_{ii}+E_{0}k_{1}G_{pb}-EGP_{0}}{k_{2}} \end{array}$$ (148)

$$\begin{array}{c} U_{id0}=k_{1}G_{pb}-k_{2}G_{tb} \end{array}$$ (149)

$$\begin{array}{c} k_{ir}=\frac{U_{id0}\left(K_{m0}+G_{tb}\right)}{V_{m0}G_{tb}} \end{array}$$ (150)

$$\begin{array}{c} I_{lb}=\frac{m_{2}I_{pb}}{m_{1}+m_{3}} \end{array}$$ (151)

$$\begin{array}{c} \alpha =\frac{5}{2[Dose_{0}\left(1-b\right)]} \end{array}$$ (152)

$$\begin{array}{c} \beta =\frac{5}{2(Dose_{0}c)} \end{array}$$ (153)

$$\begin{array}{c} k_{empt0}=k_{min0}+\frac{k_{max0}-k_{min0}}{2}*\left\{tanh\left[\alpha *\left(-b*Dose_{0}\right)\right]-tanh\left[\beta *\left(-c*Dose_{0}\right)\right]+2\right\} \end{array}$$ (154)

$$\begin{array}{c} f_{2}=log\left(G_{th}^{r_{2}}\right)-log\left(G_{b}^{r_{2}}\right) \end{array}$$ (155)

$$\begin{array}{c} I_{0}^{\prime }=I_{b} \end{array}$$ (156)

$$\begin{array}{c} I_{sc1ss}=\frac{u_{sc0}}{k_{d}+k_{a1}} \end{array}$$ (157)

$$\begin{array}{c} I_{sc2ss}=\frac{\frac{k_{d}}{k_{a}2}}{I_{sc1ss}} \end{array}$$ (158)

$$\begin{array}{c} G_{sc0}=G_{b} \end{array}$$ (159)

$$\begin{array}{c} Ra_{Isc0}=k_{a1}I_{sc1ss}+k_{a2}I_{sc2ss} \end{array}$$ (160)

$$\begin{array}{c} Ra_{Iih0}=k_{aIih}I_{ih0} \end{array}$$ (161)

$$\begin{array}{c} Ra_{I0}=Ra_{Isc0}+Ra_{Iih0} \end{array}$$ (162)

$$\begin{array}{c} SR_{H}^{b}=nH_{gon} \end{array}$$ (163)

$$\begin{array}{c} SR_{Hb}^{S}=SR_{H}^{b} \end{array}$$ (164)

### The Hovorka Model

**Amount of glucose**
$$\begin{array}{c} \begin{array}{cc} \frac{dQ_{1}}{dt} & =U_{G}\left(t\right)-x_{1}\left(t\right)Q_{1}\left(t\right)-F_{01}^{c}\left(t\right)-F_{R}\left(t\right)+\\ & +k_{12}Q_{2}\left(t\right)+EGP0\left[1-x_{3}\left(t\right)\right] \end{array} \end{array}$$ (165)
$$\begin{array}{c} \frac{dQ_{2}}{dt}=x_{1}\left(t\right)Q_{1}\left(t\right)-\left[k_{12}+x_{2}\left(t\right)\right]Q_{2}\left(t\right) \end{array}$$ (166)
**Measurable blood glucose concentration**
$$\begin{array}{c} G\left(t\right)=\frac{Q_{1}\left(t\right)}{V_{G}} \end{array}$$ (167)
**Glucose utilization by the central nervous system**
$$\begin{array}{c} F_{01}^{c}=\{\begin{array}{cc} F_{01} & G(t)\geq 4.5mmol/L\\ F_{01}\frac{G(t)}{4.5} & otherwise \end{array} \end{array}$$ (168)
**Renal excretion of glucose**
$$\begin{array}{c} F_{R}=\{\begin{array}{cc} 0.003\left[G\left(t\right)-9\right]V_{G} & G(t)\geq 9mmol/L\\ 0 & otherwise \end{array} \end{array}$$ (169)
**Insulin effect on distribution/transport of glucose**
$$\begin{array}{c} \frac{dx_{1}}{dt}=-k_{a1}x_{1}\left(t\right)+k_{b1}I\left(t\right) \end{array}$$ (170)
**Insulin effect on glucose disposal**
$$\begin{array}{c} \frac{dx_{2}}{dt}=-k_{a2}x_{2}\left(t\right)+k_{b2}I\left(t\right) \end{array}$$ (171)
**Insulin effect on EGP released from liver**
$$\begin{array}{c} \frac{dx_{3}}{dt}=-k_{a3}x_{3}\left(t\right)+k_{b3}I\left(t\right) \end{array}$$ (172)
**Oral CHO intake expressed as glucose equivalent**
  $$\begin{array}{c} D=frac1000M_{wG}d\left(t\right) \end{array}$$ (173)
  With $M_{wG}=[\frac{g}{mol}]$ glucose molecular weight
**Glucose in the absorption compartment**
$$\begin{array}{c} \frac{dD_{1}}{dt}=A_{G}D\left(t\right)-\frac{1}{\tau_{D}}D_{1}\left(t\right) \end{array}$$ (174)
**Glucose in the conversion compartment**
$$\begin{array}{c} \frac{dD_{2}}{dt}=\frac{1}{\tau_{D}}D_{1}\left(t\right)-\frac{1}{\tau_{D}}D_{2}\left(t\right) \end{array}$$ (175)
**The glucose absorption rate**
$$\begin{array}{c} U_{G}\left(t\right)=\frac{D_{2}\left(t\right)}{\tau_{D}} \end{array}$$ (176)
**Amount of short-acting insulin**
$$\begin{array}{c} \frac{dS_{1}}{dt}=u\left(t\right)-\frac{1}{\tau_{S}}S_{1}\left(t\right) \end{array}$$ (177)
$$\begin{array}{c} \frac{dS_{2}}{dt}=\frac{1}{\tau_{S}}S_{1}\left(t\right)-\frac{1}{\tau_{S}}S_{2}\left(t\right) \end{array}$$ (178)
**Insulin concentration**
$$\begin{array}{c} \frac{dI}{dt}=\frac{U_{I}}{V_{I}}-k_{e}I\left(t\right) \end{array}$$ (179)
**Insulin absorption rate into the blood**
$$\begin{array}{c} \frac{dU_{I}}{dt}=\frac{S_{2}}{\tau_{S}} \end{array}$$ (180)

#### Determined parameters

$$\begin{array}{c} V_{G}=0.16*B_{oW} \end{array}$$ (181)

$$\begin{array}{c} V_{I}=0.12*B_{oW} \end{array}$$ (182)

$$\begin{array}{c} EGP_{0}=0.161*B_{oW} \end{array}$$ (183)

$$\begin{array}{c} Q_{10}=G_{0}V_{G} \end{array}$$ (184)

$$\begin{array}{c} F_{R0}=\{\begin{array}{cc} 0.003\left[G_{0}-9\right]V_{G} & G_{0}\geq 9mmol/L\\ 0 & otherwise \end{array} \end{array}$$ (185)

$$\begin{array}{c} D_{10}=D_{0}A_{G}\tau_{D} \end{array}$$ (186)

$$\begin{array}{c} D_{20}=D_{10} \end{array}$$ (187)

$$\begin{array}{c} S_{10}=u_{is0}\tau_{S} \end{array}$$ (188)

$$\begin{array}{c} S_{20}=S_{10} \end{array}$$ (189)

$$\begin{array}{c} U_{I0}=\frac{S_{20}}{\tau_{S}} \end{array}$$ (190)

$$\begin{array}{c} I_{0}=\frac{U_{I0}}{k_{e}V_{I}} \end{array}$$ (191)

$$\begin{array}{c} U_{G0}=\frac{D_{20}}{\tau_{D}} \end{array}$$ (192)

$$\begin{array}{c} x_{10}=\frac{k_{b1}}{k_{a1}}I_{0} \end{array}$$ (193)

$$\begin{array}{c} x_{20}=\frac{k_{b2}}{k_{a2}}I_{0} \end{array}$$ (194)

$$\begin{array}{c} x_{30}=\frac{k_{b3}}{k_{a3}}I_{0} \end{array}$$ (195)

$$\begin{array}{c} Q_{20}=Q_{10}\frac{x_{10}}{x_{20}+k_{12}} \end{array}$$ (196)

$$\begin{array}{c} F_{010}^{c}=\{\begin{array}{cc} F_{01} & G_{0}\geq 4.5mmol/L\\ F_{01}\frac{G_{0}}{4.5} & otherwise \end{array} \end{array}$$ (197)

### T1DM Sorensen model initialization

**Arterial Glucose Concentration Cycle**

*G*_*H*0_ = [Guess Arterial Glucose Concentration]

**Initialize Glucagon model**

compute $M_{P\Gamma R0}^{I}$, $M_{P\Gamma R0}^{G}$

$\Gamma_{0}^{N}=M_{P\Gamma R0}^{I}M_{P\Gamma R0}^{G}$

**Initialize Glucose model**

$G_{BV0}=G_{H0}-\frac{r_{BGU0}}{Q_{B}^{G}}$

$G_{J0}=G_{H0}-\frac{r_{JGU}}{Q_{J}^{G}}$

compute $M_{PGU0}^{I}$

$G_{PV0}=\frac{G_{H0}}{1+\frac{V_{PI}M_{PGU0}^{I}r_{PGU}^{B}}{Q_{P}^{G}V_{PI}G_{PI}^{B}+T_{P}^{G}Q_{P}^{G}M_{PGU0}^{I}r_{PGU}^{B}}}$

**Kidney Glucose Concentration Cycle**

*G*_*K*0_ = [Guess Kidney Glucose Concentration]

compute *r*_*KGE*0_

verify that $G_{K0^{\prime }}=G_{H0}-\frac{r_{KGE0}}{Q_{K}^{G}}$

is the same of *G*_*K*0_, otherwise re-start from [Kidney Glucose Concentration Cycle]

**END Kidney Glucose Concentration Cycle**

**Liver Glucose Concentration Cycle**

*G*_*L*0_ = [Guess Liver Glucose Concentration]

compute *r*_*HGP*0_, *r*_*HGU*00_

verify that $G_{L0^{\prime }}=\frac{1}{Q_{L}^{G}}\left(Q_{A}^{G}G_{H0}+Q_{J}^{G}G_{J0}+r_{HGP0}-r_{HGU0}\right)$

is the same of *G*_*L*0_, otherwise re-start from [Liver Glucose Concentration Cycle]

**END Liver Glucose Concentration Cycle**

verify that $G_{H0^{\prime }}=\frac{1}{Q_{H}^{G}}\left(Q_{B}^{G}G_{BV0}+Q_{L}^{G}G_{L0}+Q_{K}^{G}G_{K0}+Q_{P}^{G}G_{PV0}-r_{RBCU}\right)$

is the same of *G*_*H*0_, otherwise re-start from [Arterial Glucose Concentration Cycle]

**END Arterial Glucose Concentration Cycle**

$G_{PI0}=\frac{G_{PV0}}{1+\frac{M_{PGU}^{I}r_{PGU}^{B}T_{P}^{G}}{V_{PI}G_{PI0}^{B}}}$

$G_{BI0}=G_{BV0}-\frac{T_{B}r_{BGU}}{V_{BI}}$

Metabolism: initialize using values computed on last glucose model mass balance iteration

compute: $M_{HGP0}^{I}$,$M_{HGU0}^{I}$

$f_{2}=\frac{M^{\Gamma_{0}}-1}{2}$

## References

[1] Guyton C, Hall E. Textbook of Medical Physiology Eleventh Edition. Elsevier Saunders; 2006.

[2] Bergman RN, Ider YZ, Bowden CR, Cobelli C. Quantitative estimation of insulin sensitivity. American Journal of Physiology. 1979;236:667–677. 443421 10.1152/ajpendo.1979.236.6.E667

[3] Chiang JL, Kirkman MS, Laffel MBL, Peters AL. Type 1 Diabetes Through the Life Span: A Position Statement of the American Diabetes Association. Diabetes Care. 2014;36:2034–2054. doi: 10.2337/dc14-1140 24935775 PMC5865481

[4] WHO. Gobal report on diabetes. World Health Organization; 2016.

[5] HHS. National Diabetes Statistics Report 2020 Estimates of Diabetes and Its Burden in the United States. U.S. Department of Health and Human Services; 2020.

[6] DeFronzo RA, Bonadonna RC, Ferrannini E. Pathogenesis of NIDDM. A balanced overview. Diabetes Care. 1992;15(7):318–368. doi: 10.2337/diacare.15.3.318 1532777

[7] ADA. Standards of Medical Care in Diabetes—2010. Diabetes Care. 2010;33:11–61. doi: 10.2337/dc10-S011 PMC2797382 20042772

[8] Marín-Peñ alver JJ, Martín-Timón I, Sevillano-Collantes C, Cañizo Gómez FJ. Update on the treatment of type 2 diabetes mellitus. World J Diabetes. 2016;7(7):354–395. 27660695 10.4239/wjd.v7.i17.354 PMC5027002

[9] KAISER AB, ZHANG N, DER PLUIJM WV. Global Prevalence of Type 2 Diabetes over the Next Ten Years (2018-2028). Diabetes. 2018;67(Supplement 1). doi: 10.2337/db18-202-LB

[10] Cobelli C, Renard E, Kovatchev B. Artificial Pancreas: Past, Present, Future. Diabetes. 2011;60:2672–282. doi: 10.2337/db11-0654 22025773 PMC3198099

[11] Doyle FJ III, Huyett LM, Lee JB, Zisser HC, Dassau E. Closed-Loop Artificial Pancreas Systems: Engineering the Algorithms. Diabetes Care. 2014;37:1191–1197. doi: 10.2337/dc13-2108 24757226 PMC3994938

[12] Peyser T, Dassau E, Breton M, Skyler S. The artificial pancreas: current status and future prospects in the management of diabetes. Ann NY Acad Sci. 2014;1311:102–123. doi: 10.1111/nyas.12431 24725149

[13] Steil GM, Rebrin K. Closed-loop insulin delivery—what lies between where we are and where we are going? Asheley Publications. 2005;2:353–362. 10.1517/17425247.2.2.353 16296759

[14] Kovács L, Benyó B, Bokor J, Benyó Z. Induced *L*_2_-norm minimization of glucose-insulin system for Typed I diabetic patients. Computer Methods and Programs in Biomedicine. 2011;102:105–118. doi: 10.1016/j.cmpb.2010.06.019 20674065

[15] Owens C, Zisser H, Jovanovic L, Srinivasan B, Bonvin D, Doyle FJ III. Run-to-Run Control of Blood Glucose Concentrations for People With Type 1 Diabetes Mellitus. Biomedical Engineering. 2006;53(12):996–1005. 16761826 10.1109/TBME.2006.872818

[16] Kovatchev B, Breton M, Dalla Man C, Cobelli C. In silico preclinical trials: a proof of concept in closed-loop control of type 1 diabetes. Journal Diabetes Science Technology. 2009;3:44–55. doi: 10.1177/193229680900300106 19444330 PMC2681269

[17] Hovorka R, Shojaee-Moradie F, Carroll PV, Chassin LJ, Gowrie JI, Jackson RST, et al. Partitioning glucose distribution/transport, disposal, and endogenous production during IVGTT. Am J Physiol Endocrinol Metab. 2002;282:992–1007. doi: 10.1152/ajpendo.00304.2001 11934663

[18] Hovorka R, Canonico V, Chassin LJ, Haueter U, Massi-Benedetti M, Federici MO, et al. Nonlinear model predictive control of glucose concentration in subjects with type 1 diabetes. Institute of Physics Publishing. 2004; p. 905–920. 15382830 10.1088/0967-3334/25/4/010

[19] Hovorka R. Closed-loop insulin delivery from bench to clinical practice. Nature Reviews Endocrinology. 2011;7:385–395. doi: 10.1038/nrendo.2011.32 21343892

[20] Sorensen JT. A Physiologic Model of Glucose Metabolism in Man and Its Use to Design and Improved Insulin Therapies for Diabetes. Massachussets Institute of Technology; 1985.

[21] Chee F, Fernando T. Closed-Loop Control of Blood Glucose. Springer; 2007. 10.1177/0310057X0203000306 12075636

[22] Dalla Man C, Rizza RA, Cobelli C, Fellow I. Meal Simulation Model of the Glucose-Insulin System. IEEE TRANSACTIONS ON BIOMEDICAL ENGINEERING. 2007;54(10):1740–1749. doi: 10.1109/TBME.2007.893506 17926672

[23] Dalla Man C, Micheletto F, Lv D, Breton M, Kovatchev B, Cobelli C. The UVAPadova Type 1 Diabetes Simulator: New Features. Journal of Diabetes Science and Technology. 2014;8:26–34. doi: 10.1177/1932296813514502 24876534 PMC4454102

[24] Visentin R, Campos-Náñez E, Schiavon M, Lv D, Vettoretti M, Breton M, et al. The UVAPadova Type I Diabetes Simulator Goes From Single Meal to Single Day. Journal of Diabetes Science and Technology. 2018;12:273–281. doi: 10.1177/1932296818757747 29451021 PMC5851236

[25] Dalla Man C, Raimondo DM, Rizza RA, Cobelli C. GIM, Simulation Software of Meal Glucose–Insulin Model. Journal of Diabetes Science and Technology. 2007;1:323–330. 19885087 10.1177/193229680700100303 PMC2769591

[26] Visentin R, Giegerich MS, Jäger R, Dahmen R, Boss A, Grant M, et al. Improving Efficacy of Inhaled Technosphere Insulin (Afrezza) by Postmeal Dosing: In-silico Clinical Trial with the University of Virginia/Padova Type 1 Diabetes Simulator. Diabetes Technology & Therapeutics. 2016;18(9):574–585. doi: 10.1089/dia.2016.0128 27333446 PMC5035370

[27] Visentin R, Dalla Man C, Cobelli C, Fellow I. One-Day Bayesian Cloning of Type 1 Diabetes Subjects: Towards a Single-Day UVAPadova Type 1 Diabetes Simulator. IEEE Trans Biomed Eng. 2016;63:2416–2424. 26930671 10.1109/TBME.2016.2535241

[28] Panunzi S, Pompa M, Borri A, Piemonte V, De Gaetano A. A revised Sorensen model: Simulating glycemic and insulinemic response to oral and intra-venous glucose load. PLOS ONE. 2020;15:1–30. doi: 10.1371/journal.pone.0237215 32797106 PMC7428140

[29] Lv D, Kulkarni SD, A C, Keith S, Pettis R, Kovatchev BP, et al. Pharmacokinetic Model of the Transport of Fast-Acting Insulin From the Subcutaneous and Intradermal Spaces to Blood. Journal of Diabetes Science and Technology. 2015;9:831–840. doi: 10.1177/1932296815573864 25759184 PMC4525663

[30] Hinshaw L, Dalla Man C, Nandy DK, Saad A, Bharucha AE, Levine AJ, et al. Pathogenesis of NIDDM. A balanced overview. Diabetes. 2013;62:2223–2229. 23447123 10.2337/db12-1759 PMC3712033

